# Detection of Reproducible Major Effect QTL for Petal Traits in Garden Roses

**DOI:** 10.3390/plants10050897

**Published:** 2021-04-29

**Authors:** Dietmar Schulz, Marcus Linde, Thomas Debener

**Affiliations:** Institute of Plant Genetics, Molecular Plant Breeding Section, Leibniz University Hannover, Herrenhäuser Straße 2, 30419 Hannover, Germany; schulz@genetik.uni-hannover.de (D.S.); linde@genetik.uni-hannover.de (M.L.)

**Keywords:** SNP, KASP, fragrance, petal size, petal number, association mapping, marker-assisted selection, flower QTL

## Abstract

The detection of QTL by association genetics depends on the genetic architecture of the trait under study, the size and structure of the investigated population and the availability of phenotypic and marker data of sufficient quality and quantity. In roses, we previously demonstrated that major QTL could already be detected in small association panels. In this study, we analyzed petal number, petal size and fragrance in a small panel of 95 mostly tetraploid garden rose genotypes. After genotyping the panel with the 68 K Axiom WagRhSNP chip we detected major QTL for all three traits. Each trait was significantly influenced by several genomic regions. Some of the QTL span genomic regions that comprise several candidate genes. Selected markers from some of these regions were converted into KASP markers and were validated in independent populations of up to 282 garden rose genotypes. These markers demonstrate the robustness of the detected effects independent of the set of genotypes analyzed. Furthermore, the markers can serve as tools for marker-assisted breeding in garden roses. Over an extended timeframe, they may be used as a starting point for the isolation of the genes underlying the QTL.

## 1. Introduction

Floriculture is one of the most economically important sectors of horticultural business. Among the traits that determine the success of floricultural products, the morphological and physiological characteristics of flowers as for example the colour, the fragrance and the structure of the flower (petal number, flower size, shape) are among the most important traits. In general, shape, color, vase life and fragrance are prominent criteria that influence consumer preferences [1]. Especially in roses, which are among the most important ornamental crops, floral traits dominate the important characters for all types of use: from garden roses to cut roses to pot roses. The aesthetic features of rose flowers are of central importance for the ornamental quality of rose cultivars; therefore, commercial breeding gives special attention to floral characteristics. Flower traits, e.g., the number and color of petals, were among the first traits investigated in genetic studies [2,3]. In addition, a large number of studies have also addressed the basic aspects of unique features of the rose flower, e.g., the unique composition of scent metabolites that make up the rose fragrance [4,5,6,7,8,9,10].

Roses are highly diverse in many morphological and physiological characteristics and display high morphological and physiological diversity in flower morphology. This diversity can be exploited for genetic studies on the inheritance of these traits by association mapping, which is a population-based analysis used to identify markers that are tightly linked to the trait of interest based on the decay of linkage disequilibrium (LD) [11]. Association studies using unrelated populations provide two main advantages compared with QTL mapping: a higher resolution of the mapping population and the detection of more alleles per locus due to the higher rate of historic recombination events and the higher diversity of the mapping population compared with biparental progenies. Association mapping has the potential to discover functional genes that can be directly used in breeding. The localized functional SNPs can be converted into KASP markers and, after validation, used in independent populations to subsequently genotype and select parents for desired crosses. To reduce false positive associations [12], population structure and kinship within the association panel have to be analyzed and considered in statistical procedures as cofactors [13].

For roses, a number of genomes [14,15] have been published, and the WagRhSNP 68k Axiom SNP array [16], providing 68,893 SNPs, allows high-density marker analyses and the identification of candidate genes. GWAS has been used to determine QTL associated with anthocyanin and carotenoid contents of petals in roses [17], with adventitious shoot regeneration [18], with petal numbers [14] and the genetic analysis of callus formation [19]. In the current paper, we present markers on QTL for petal number, petal length and fragrance evaluated in the same rose panel used in previous studies by Schulz et al. [17] and Nguyen et al. [18,19].

## 2. Results

**Figure 1:**
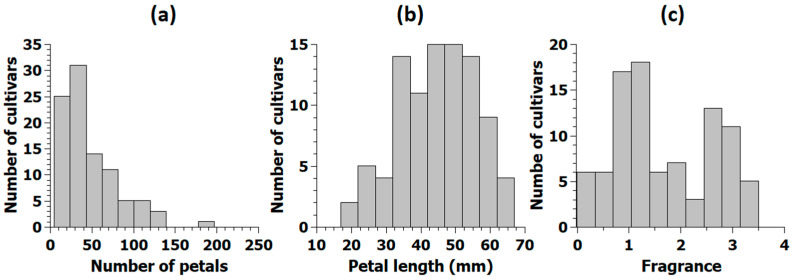
Distribution of the mean for petal number (**a**), petal length (**b**) and of the median for fragrance (**c**) in the association panel. Normal distribution was tested with Shapiro-Wilk for petal number (*p* = 0.29), petal length (*p* = 2.9 × 10^−7^) and fragrance (*p* = 2.6 × 10^−5^). Fragrance score: 0 = no fragrance, 1 = mild to moderate fragrance, 2 = good fragrance, 3 = strong fragrance, and 4 = very strong fragrance.

Measurement of petal number, petal size and fragrance among 95 garden roses of the association panel displayed considerable quantitative variation, which differed in terms of the shape of the distribution among the three traits (Figure 1). The number of individuals of the validation panel varied for the traits, as only individuals with properly scored KASP markers were phenotyped. Individuals of the validation panel were arbitrarily selected from a pool of DNA samples from previous projects available at Leibniz University Hannover and phenotyped afterwards.

### 2.1. SNP-Markers Associated with Petal Number

The distribution of petal numbers among the 95 cultivars of the association panel was skewed to the left with a minimum of 5.1 petals for cultivar ‘Juanita’ and a maximum of 197 petals for ‘Perpetually Yours’ and an overall mean of 53 petals (Figure 1a). Among genotypes of the validation panel, a minimum average number of five petals were observed for 17 cultivars, and a maximum of 132.5 petals could be reported for cultivar ‘Zaide’. The overall mean in the validation panel was 38.2 petals, and the distribution was strongly left skewed (Supplementary Figure S1a).

**Figure 2:**
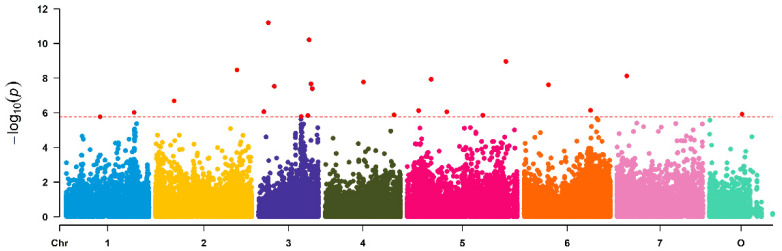
Manhattan plot of associations between 63,000 SNP markers and petal number in a set of 95 garden roses. For a better view of the clusters, the *y*-axis was scaled only to 1 × 10^−12^. Supplementary Figure S2 displays the full Manhattan plot including the outlier markers. The *x*-axis represents the chromosomes of *Rosa chinensis*, including chromosome 0 with contigs not assigned to a precise location to date. Associations were calculated in Tassel 3.0 using MLM including population structure (Q), kinship (K) and Box-Cox transformed data of fragrance. The red dotted line indicates the Bonferroni corrected level of significance (−log10 = 5.75).

Association analyses with SNP data from the SNP array and the MLM method in TASSEL [20] revealed 46 significantly associated SNPs distributed over all chromosomes (Figure 2 and Supplementary Figure S2). Some of these formed distinct peaks on chromosomes 1, 3, 5 and 6.

**Figure 3:**
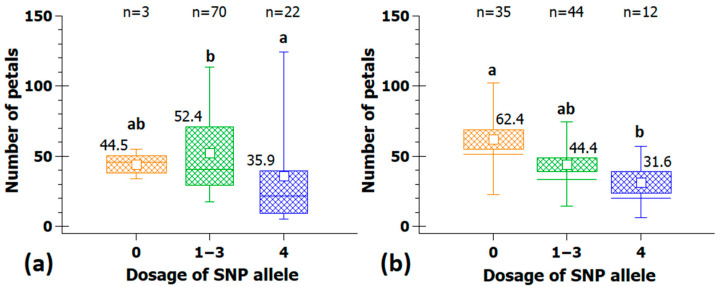
SNP-markers with strong effects on petal number. (**a**) Effect of SNP-marker Rh_PN_SNP28 (chr5; 9 Mbp) and (**b**) effect of SNP-marker Rh_PN_SNP40 (chr6; 52.0 Mbp) which could only be analyzed in 91 individuals. Numbers on the *x*-axis show the allelic dosage for the four marker classes (0 and 4 for the alternative homozygotes, and 1–3 for the heterozygotes, which were combined into one group for technical reasons). For each allele dosage group, the number of individuals (n) is given on top; groups that are significantly different at *p* ≤ 0.05 are indicated by letters above the whiskers (Kruskal-Wallis analysis of variance on ranks with all pairwise multiple comparisons (Dunn’s method); mean values are given above the box.

Two closely linked peaks on chromosome 3 (at 29.0 Mb and 33.3 Mb) have been reported earlier, and markers from one of the peaks have also been validated in independent populations [14] and will not be discussed further here. We focused on markers from peaks on chromosomes 1, 5 and 6. The peak on chromosome 5 is between 8.4 and 9.0 Mb and a detailed analysis of the marker Rh_PN_SNP28 (*p* = 7.82 × 10^−7^) at that locus showed an effect size of 16.2 petals between dosage group 4 and the remaining groups (Figure 3a). On chromosome 6, two peaks were located, and a significantly associated marker from the peak at 52.0 Mb (marker Rh_PN_SNP40, *p* = 7.32 × 10^−7^) displayed an effect size of 30.8 petals (Figure 3b), while the second marker from the peak at 57.0 Mb (marker Rh_PN_SNP41, *p* = 2.15 × 10^−6^) had an effect of 21.7 petals.

**Figure 4:**
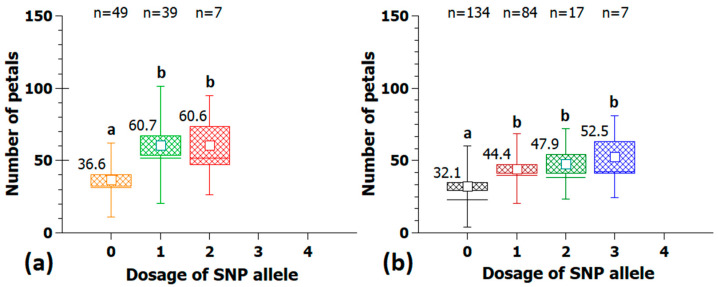
(**a**) Effect of KASP-marker Rh_PN_SNP6K (chr1; 53.2 Mbp) on petal number in the association panel and (**b**) validation of the same marker with 242 independent cultivars. Shown are the number of individuals in each group and the average number of petals/flower. Letters above the whiskers mark groups that are significantly different at *p* ≤ 0.05 (Kruskal-Wallis analysis of variance on ranks with all pairwise multiple comparisons (Dunn’s method).

The marker Rh_PN_SNP6 (*p* = 9.21 × 10^−7^) from the peak on chromosome 1 at 53.2 Mbp is linked to a locus with an effect size of 24 petals. This marker was converted into a KASP marker and tested in 242 independent cultivars. Cultivars with dosages of 0 had a mean 20.4 petals less than cultivars with a dosage of 3 for the SNP allele (Figure 4).

### 2.2. SNP-Markers Associated with Petal Length

Petal length was normally distributed in both the association panel (Figure 1b) and the validation population with 179 individuals (Supplementary Figure S1b). The mean value for petal length was 45.3 mm for the 96 individuals of the association panel and 33.8 mm for the validation panel.

**Figure 5:**
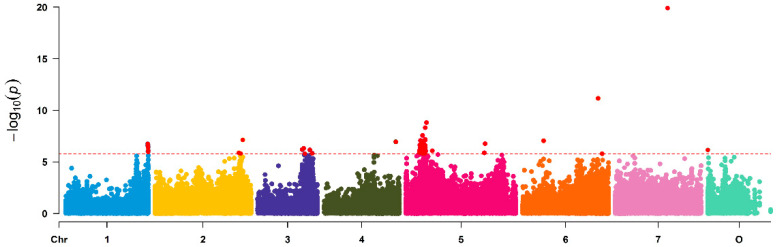
Manhattan plot of associations between 63,000 SNP markers and petal length in a set of 95 garden roses. The *x*-axis represents the chromosomes of *Rosa chinensis*, including chromosome 0 with contigs not assigned to a precise location to date. Associations were calculated in Tassel 3.0 using MLM including population structure (Q) and kinship (K) and Box-Cox transformed data of fragrance. The red dotted line indicates the Bonferroni corrected level of significance (−log10 = 5.75).

Analyses of marker trait associations revealed 68 significantly associated SNPs on all chromosomes and distinct peaks on chromosomes 1, 2, 3 and 5, as well as on chromosome 0, as shown in Figure 5.

**Figure 6:**
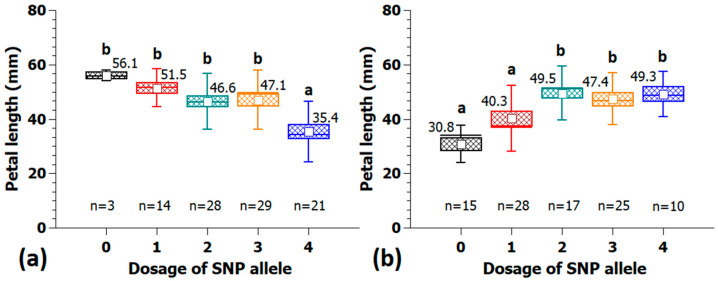
Example of two SNP markers with large effects on petal length located on chr1 at 64.5 Mbp (Rh_PL_SNP6) (**a**) and on chr3 at 36.0 Mbp (Rh_PL_SNP16) (**b**). Neither marker has been validated in independent populations. On the *x*-axis are the allelic dosages for the four marker classes (0 and 4 for the homozygote classes and 1–3 for the heterozygotes), and on the *y*-axis, the petal length. Per allele dosage group, the number of individuals (n) is given on the bottom; groups that are significantly different at *p* ≤ 0.05 are indicated by letters above the whiskers. Tests were performed by ANOVA followed by all pairwise multiple comparison procedures (Holm-Sidak method). Small white square = mean; continuous line = median; box = standard error; and whisker = standard deviation.

**Figure 7:**
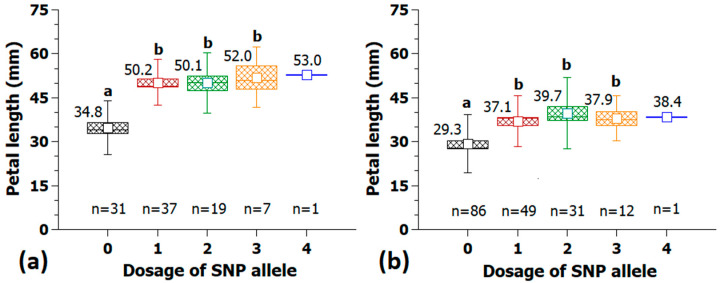
Validation of the KASP marker Rh_PL_SNP49K from the peak at 14.5 Mbp on chr5 for petal length with 95 cultivars in the association panel (**a**) and 179 independent tetraploid cultivars (**b**). On the *x*-axis are the allelic dosages for the four marker classes (0 and 4 for the homozygote classes and 1–3 for the heterozygotes), and on the *y*-axis, the petal length. Per allele dosage group, the number of individuals (n) is given on the bottom; groups that are significantly different at *p* ≤ 0.05 are indicated by letters above the whiskers (Kruskal-Wallis rank sum test); mean values are given above the box. Small white square = mean; continuous line = median; box = standard error; and whisker = standard deviation.

The effect of different SNP markers on petal length in the association panel is shown in Figure 6 and Figure 7a. A selection of SNP markers from peaks on chr1, 3 and 5 were analyzed for their effects on petal size, which turned out to be substantial. The effect of the SNP marker Rh_PL_SNP6 on chr1 was 20.7 mm, measured as the difference in the mean in both homozygous allele groups with a continuous decrease in petal length from allele dosages zero to four (Figure 6a). The observed effect of the SNP marker Rh_PL_SNP16 on chr3 was 18.5 mm (Figure 6b).

The SNP marker Rh_PL_SNP49 located on chr5 was converted to a KASP marker and was used both in the association and validation populations. The effect in the association panel was 17.2 mm between allele dosages zero and three (Figure 7a). The quadruplex allele configuration was found in only one genotype and was therefore not useful for the calculation of effect sizes. Furthermore, a similar expression of petal length among allele dosages 1 to 3 indicates a dominant effect of the underlying SNP allele. The marker was validated in an independent population of 179 cultivars (Figure 7b) and displayed a similar dominant pattern of inheritance with a slightly smaller effect size of 10.1 mm.

### 2.3. SNP-Markers Associated with Fragrance

Fragrance was scored by sniffing harvested and bagged flowers, and fragrance intensity was rated with intensity scores from 0 (no fragrance) to 4 (strong fragrance). For validation of the markers, a simpler score ranging from 1 (no fragrance) to 3 (strong fragrance) was used, as preexisting scores in the range from 1 to 3 were already available at the German Variety Protection Office. Scores for fragrance displayed two maxima and were not normally distributed in the association panel (Figure 1c). In the association panel, 26 of the cultivars displayed mean values between zero and one, indicating none to mild fragrance. In contrast, 12 cultivars had strong to very strong fragrance (scores 3 to 4); among them the cultivar ‘Blue River’ with the strongest scent in the panel.

**Figure 8:**
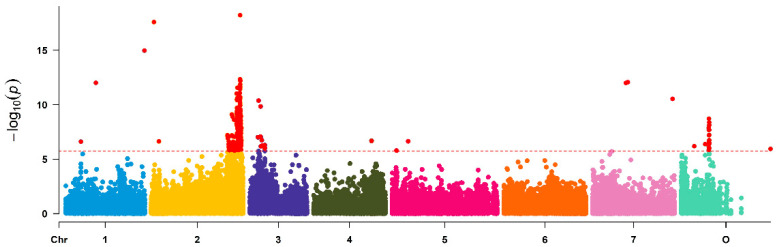
Manhattan plot of associations between 63,000 SNP markers and fragrance in a set of 95 garden roses. For a better view of the clusters, the *y*-axis was scaled only to 1 × 10^−20^. Supplementary Figure S3 displays the full Manhattan plot including the outlier markers. The *x*-axis represents the chromosomes of *Rosa chinensis*, including chromosome 0 with contigs not assigned to a precise location to date. Associations were calculated in Tassel 3.0 using MLM, including population structure (Q) and kinship (K), and box Cox transformed fragrance data. The red dotted line indicates the Bonferroni corrected level of significance (-log10 = 5.75).

The analysis of associated SNPs revealed the highest number among the three traits analyzed in this study. A total of 218 significantly associated SNPs were found on all chromosomes except chromosome 6 (Figure 8 and Supplementary Figure S3).

The largest cluster with 172 SNPs was located on chr2 in a region from 62.3 Mbp to 73.7 Mbp. Other prominent peaks were detected on chromosomes 1, 3, 4 and 0. Seven SNPs were detected on chr7, four on chr5 and only one on chr4. On chr0, we detected 19 significant SNPs with a clear peak at 22 Mbp, and nine SNPs were located on chr3 in a region around 9 Mbp.

**Figure 9:**
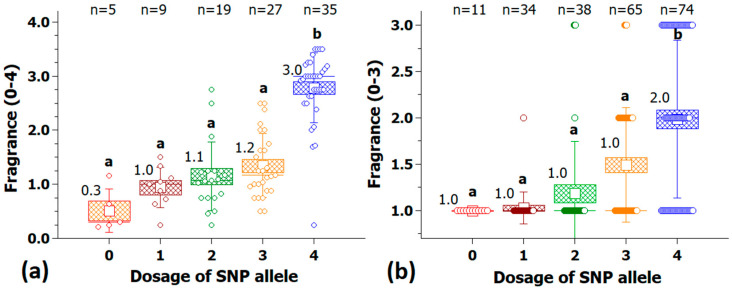
Validation of the KASP marker Rh_FR_SNP139K (Chr 2, 72.49 Mbp) for fragrance with 95 cultivars in the association panel (**a**) and with 222 independent tetraploid cultivars (**b**). On the *x*-axis are the allelic dosages for the four marker classes (0 and 4 for the homozygote classes and 1–3 for the heterozygotes), and on the *y*-axis, the fragrance score. For each allele dosage group, the number of individuals (n) is given on top; groups that are significantly different at *p* ≤ 0.05 are indicated by letters above the whiskers (Kruskal-Wallis rank sum test); median values are given above the box. Small white square = mean; continuous line = median; box = standard error; and whisker = standard deviation.

**Figure 10:**
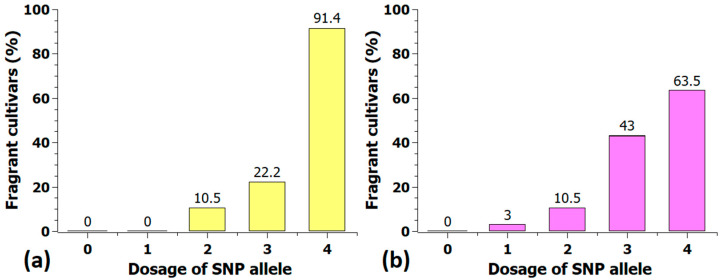
Percentage of fragrant cultivars (fragrance score ≥ 2) in the five marker classes of the KASP-marker Rh_FR_SNP139K in the association panel (**a**) and in the validation population with 222 cultivars (**b**). Shown above the columns are the percentages of fragrant cultivars.

A selection of markers from different chromosomes at peak regions with a strong association with fragrance were analyzed for their effect size (Supplementary Table S11). Some of these markers were also converted into KASP markers, and the association panel was retested to validate the technical use of these markers and to confirm the effect in independent populations. With the KASP marker Rh_FR_SNP139K (chr2, 72.5 Mbp), a clear difference in fragrance between the two homozygous groups was visible, with median values of 0.3 and 3.0 (Figure 9a). A similar effect was found for the validation with 222 cultivars (Figure 9b). The median increased significantly from one to two in the group with the ‘positive homozygous allele’. The percentage of cultivars with a fragrance score ≥2 in the five marker classes is shown in Figure 10, indicating that 91% of the cultivars with a quadruplex configuration had a strong scent.

**Figure 11:**
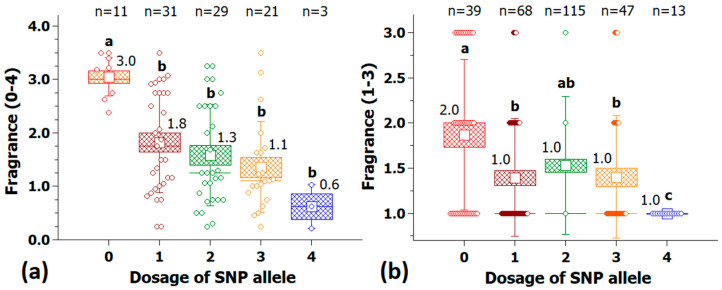
Validation of the KASP marker Rh_FR_SNP201K (Chr 3, 7.3 Mbp) for fragrance with 95 cultivars in the association panel (**a**) and with 282 independent tetraploid cultivars (**b**). On the *x*-axis are the allelic dosages for the four marker classes (0 and 4 for the homozygote classes and 1–3 for the heterozygotes), and on the *y*-axis, the fragrance score. For each allele dosage group, the number of individuals (n) is given on top; groups that are significantly different at *p* ≤ 0.05 are indicated by letters above the whiskers (Kruskal-Wallis rank sum test); median values are given above the box. Small white square = mean; continuous line = median; box = standard error; and whisker = standard deviation.

The second KASP marker for fragrance, Rh_FR_SNP201K, is located on chr3. The group of plants with a marker score of 0 (nulliplex) contains only 11 cultivars, but each of them has a strong fragrance (Figure 11a). The validation with 282 independent cultivars was also successful, but the group with the positive allele dosage nulliplex also contained ‘none to mild’ scented cultivars (Figure 11b). The distribution of markers among the allele dosage groups indicates a dominant inheritance, with each of the alleles of dosage groups 1–4 reducing the fragrance significantly without significant differences among these groups.

**Figure 12:**
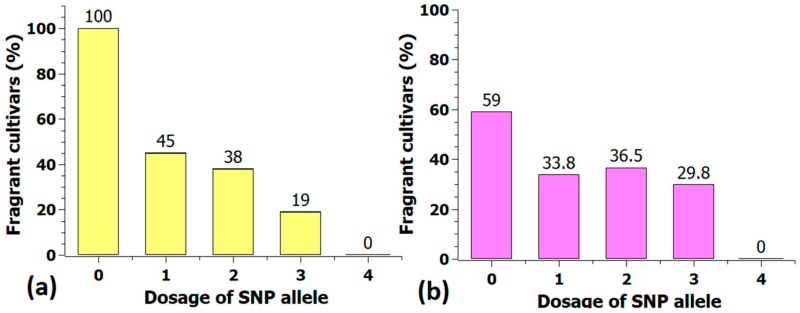
Percentage of fragrant cultivars (fragrance score ≥ 2) in the five marker classes of the KASP marker Rh_FR_SNP201K in the association panel (**a**) and in the validation population with 282 cultivars (**b**). Shown above the columns are the percentages of fragrant cultivars.

In the nulliplex group of 39 cultivars, the fragrance intensity of 23 cultivars (59%) was rated as good to strong (fragrance score ≥ 2), whereas all 13 cultivars (100%) with a quadruplex allele dosage were rated as ‘none to mild’ fragrant (Figure 12). The percentage of good to strong scented cultivars in the heterozygous group of 115 cultivars was 36.5%, exactly between the fragrant and nonfragrant homozygous groups. A similar effect was found for the KASP marker Rh_FR_SNP139K (Figure 10). From allele dosage zero to dosage four, the percentage of scented roses increased from 0% to 63.5% (47 cultivars).

## 3. Discussion

We analyzed markers associated with petal number, petal length and fragrance in roses and detected a number of associated markers, some of which were confirmed in independent populations.

The use of genetic markers is common not only in plant breeding programs for many major crops but also in basic plant research [21,22]. Markers are used for gene isolation to characterize genotypes for marker-assisted introgression of desired alleles and in a variety of protection processes. Furthermore, they are useful for marker-assisted selection of favorite traits by choosing the optimal combinations of parents and to characterize the resulting progenies. This increases the selection efficiency and shortens the time for selection of traits that cannot be evaluated in young seedlings, such as disease resistance and scent or productivity parameters. However, in ornamental plant breeding, markers have not been used to this extent for several reasons. Apart from the large number of different species used in the floriculture industry, which reduces the resources available for the individual species, the high frequency of complex polyploid genomes and other unfavorable characteristics limit marker applications in ornamental plants thus far.

In roses, markers linked to monogenic, as well as polygenic traits, have been reported for many different characteristics [23,24]. However, most of the markers reported for rose traits to date suffer from three major disadvantages: (1) They are either dominant or codominant markers (e.g., AFLPs or SSRs) that reveal very limited information about marker dosage in polyploids; (2) they have been developed for restricted gene pools mostly defined by the two parental genotypes of a biparental population and therefore, often cannot be used in genetically unrelated genotypes; and (3) analyses of linkage to the associated trait often reveal large confidence intervals due to a small number of recombination events available in biparental segregating populations and relatively low marker numbers. An example of this is one of our own publications from 2010 [8], in which single loci and QTL for scent components were mapped onto a marker map mostly based on AFLP and SSR markers.

Very few publications have recently reported markers derived from association studies that are based on a larger number of unrelated genotypes and therefore have a much broader genetic base and use a large number of molecular markers covering the genome [17,19,25,26].

In this study, we were able to detect a number of markers for loci affecting three major petal characteristics in roses. Our primary marker analyses used the WagRhSNP 68K Axiom SNP array from more than 13,000 full-length expressed genes representing 68,893 SNPs and a panel of 95 garden rose genotypes [16]. It should be mentioned that SNP markers are mostly independent of the technical platform, as they can be detected with different technologies, including high-throughput sequencing. This is important for the use of QTL information when technologies for marker detection may change to other technology platforms. SNP arrays also provide information on the marker dosage, which in some cases allowed us to detect additive marker effects, such as markers associated with petal number (Figure 4b) and dominant effects, such as markers associated with petal size (Figure 7). This is important for the interpretation of the gene action underlying the respective QTL peak. In the mostly polyploid roses, SNP platforms (SNP chips, KASP markers or amplicon sequencing) are particularly suited to generate this information for marker QTL.

Furthermore, a selection of markers, which were tested in an independent panel of more than 200 genotypes, demonstrates that these markers are not restricted to a small gene pool of roses but rather useful in a broad germplasm. Thus far, these groups mostly included garden roses; therefore, in the future, these markers should also be tested in panels including cut and pot roses to evaluate their performance in all major groups of cultivated roses.

### 3.1. Application of Markers in Rose Breeding and Research

As already outlined, marker-assisted breeding in ornamentals has not been employed compared with the agricultural sector or fruit and vegetable crops. However, even if the large-scale application of marker-assisted selection in progeny of crosses among elite clones may not be in view for roses, improvements by MAS of crossing partners could be achieved very rapidly. Here, the dosage information that can be obtained by several SNP genotyping platforms can be used to select parents with higher dosages of favorable alleles [24]. This would result in larger proportions of progeny with favorable allele combinations and thus desired characteristics from which superior individuals for other traits can be selected.

Using the KASP marker Rh_FR_SNP139K, which is associated with fragrance, one could easily select parental genotypes with the positive allele in duplex and triplex for crosses, which made up more than 60% of our validation panel, leading to progenies with 100% of the individuals having the positive allele at least in duplex. Assuming the fragrance data from our validation panel, approximately 80% of these will have medium to strong scented flowers, which is a favorite trait for consumers of garden roses.

This can also be achieved for different traits at the same time, leading to larger fractions of progeny with combinations of several favorable characteristics, for example, a higher number of progeny with large and scented double flowers, among which selection of other traits, such as resistance, can then be done. Therefore, even for highly heritable traits that can be easily selected visually early in the selection process, the MAS of superior crossing partners is advantageous.

The possibility of using these markers for the selection of parental genotypes is clearly due to their close linkage to QTL with large effects on the traits of interest. In addition to their applications in the breeding process, the markers we found associated with petal traits therefore also provide information on the genetic basis of the QTL and can be used to identify the candidate genes underlying the QTL.

### 3.2. Candidate Genes for Petal Number

For petal number, previous studies revealed two tightly linked loci on chromosome 3, close to a homologue of the AP2 gene, which most likely explains the switch from single flowers with five petals to flowers in which a homeotic transition of stamens leads to multiple petaled flowers with more than five petals [27]. However, in addition to the AP2 locus, additional loci determine the extent to which stamens are converted into petals, e.g., the number of petals in so-called ’double flowers’.

The QTL on chr5 at 8.4 Mbp is located approximately 17 kb from the homeotic MADS box gene AGAMOUS (AG). Dubois et al. [28] found that the expression of RhAG is downregulated in double flowers of cultivated roses and is concentrated in the central region of the meristem, which allows an increasing number of petals to develop. Additionally, Roman et al. [29] identified a QTL for petal number on LG2 close to AGAMOUS/MASAKO C1. Therefore, AG seems to be a good candidate gene for the QTL on chr5.

We localized orthologs of the transcription factor (TF) WUSCHEL (WUS) at position 53.12 Mbp only 37 Kb far from the significant QTL on chr1 at 53.2 Mbp. Prior studies have shown that AG interacts with WUS [30,31]. WUS activates the expression of AG in whorls 3 and 4 together with LEAFY (LFY) [31,32]. Mutation of the WUS box eliminates the ability of WUS to induce the expression of AG [33]. Hence, WUS is a realistic candidate gene regulating the petal number on chr1.

Additionally, the QTL on chr6 at 51.9 Mbp may interact with WUS. The QTL is located close to the ortholog of the transcription factor FANTASTIC FOUR 1-like (FAF). FAF proteins can also repress WUS in the organizing center of the shoot meristem [34] and therefore, may interact with the WUS orthologs on chr1. A reduced expression of WUS could result in a downregulation of AG with an impact on flower organ development [28]. The second QTL on chr6 at 57.9 Mbp is in a region where several MADS box genes are localized. Possible candidate genes in that region are MASAKO B3 (58.61 Mbp), a homologue of PISTLATA, which regulates petal and stamen development and is overexpressed during flower developmental stage 3 + 4 [35,36], and SKP1-like 1a at 57.62 Mbp [37,38] and the TF WUS (57.67 Mbp).

### 3.3. Candidate Genes for Petal Length

The genetic architecture of petal size regulation seems to be complex, as our analysis revealed a number of clearly defined peaks distributed over several chromosomes (Figure 5). This corresponds to numerous publications reporting complex networks regulating growth and cell proliferation [39,40,41,42,43,44,45].

On chr1, we found two significant loci, at 55.5 Mbp and between 64.25 Mbp to 64.7 Mbp. The first QTL colocalizes with TFs known to be responsible for organ growth, of which the rose expansin RHEXPA4 (54.61 Mbp) is known to modulate leaf growth by cell expansion [46]. Other candidate genes for petal length in this region are GROWTH REGULATING FACTOR 5 (GRF5, 64.67 Mbp) and GRF3 (63.91 Mbp). GRFs control cell division and are involved in the regulation of organ size [47], and mutations in these genes result in smaller petals in *Arabidopsis thaliana* [48].

On chr2, we found several genes that are involved in the regulation of plant growth between 65.6 and 68.7 Mbp. Expression analyses of LONGIFOLIA (67.5 Mbp) in *Arabidopsis* displayed a positive influence on cell elongation, resulting in elongated floral organs [49,50]. A well-described candidate gene is the TF NAC100 (69.02 Mbp). In *A. thaliana*, overexpression of RhNAC100 inhibited petal growth, while silencing of RhNAC100 in roses significantly increased petal size [51].

Additionally, on chr3, some interesting candidate genes were located. EXPA4 (34.6b Mbp) was also found on chr1 [46]. The TF bHLH79 located at 41.42 Mbp, known as BIGPETAL (BPEp), is directly involved in the control of petal size by reducing cell size [52,53]. The growth regulating gene RAPTOR1 isoform X2 (36.16 Mbp) is involved together with TOR (TARGET OF RAPAMYCIN) in the regulation of cell expansion [41].

BRASSINAZOLE-RESISTANT1/-SUPPRESSOR1 homolog 4 (BZR1/BES1, 8.06 Mbp) is located on chr5 at the beginning of a broader QTL from 8 Mbp to 15.5 Mbps. BZR1/BES1 is known to influence petal development, as was shown by a bzr1–1D mutant with enhanced BR signaling resulting in increased flower size [54].

### 3.4. Candidate Genes for Fragrance

We detected a large QTL region for scent on chr2. The highly significant cluster extended from 62.3 Mbp to 72.7 Mbp. Due to the large number of possible candidate genes in that region, we will focus on a few examples. We found tricyclene (E)-beta-ocimene synthase (EBOS) at 63.49 Mbp. Monoterpene (E)-ß-ocimene is one of the major components in several plant families [55,56,57] and is also described in roses [9,58].

The TF MYB104 at position 68.99 Mbp is also known as ODORANT1 (ODO1). In petunias, ODO1 regulates the production of volatile benzenoids by activating 5-enolpyruvylshikimate-3-phosphate (EPSP) synthase [59]. Fenske and Imaizumi [60] supposed that ODO1 also regulates the enzymes chorismate mutase (CM1) and phenylalanine ammonia lyase (PAL) in the floral volatile benzenoid/phenylpropanoid (FVBP) pathway in petunias. Van Moerkercke et al. [61] identified the gene encoding the ABC transporter (PhABCG1), which may facilitate transport through the membrane, as another target for ODO1 in petunias. This hypothesis was confirmed by Adebesin et al. [62], who showed that downregulation of PhABCG1 reduced the emission of volatiles. We also found several ABC transporters inside the cluster on chr2 between 70.16 Mbp to 71.2 Mbp with one significant SNP inside the sequence of one of these.

An additional candidate on chr2 is the TF EMISSION OF BENZENOIDS II (EOBII) at 72.3 Mbp. EOBI and EOBII regulate the shikimate and phenylpropanoid pathways in petunias [63]. Suppression of EOBII expression led to reduced levels of emitted volatiles such as benzaldehyde, phenylethanol, benzylbenzoate and isoeugenol [60]. Moreover, EOBII is a direct activator of ODO1 [64].

Furthermore, previous analyses in a diploid segregating population placed a single dominant gene for the emission of geranylacetate and a QTL for 2 phenyl-ethanol at the end of chromosome 2 [8]. As we did not dissect scent metabolites into individual compounds in our present study, these compounds may be part of the genetic factors contributing to the large effect QTL on chromosome 2. A next step in narrowing down the number of candidate genes could be transcriptomic and metabolomic analyses in groups of plants with both contrasting SNP dosages and trait expression levels, which might allow us to detect the common denominator among putative factors characterizing the individual peaks.

## 4. Materials and Methods

### 4.1. Plant Material

The association panel consisted of 95 mostly tetraploid rose cultivars from different breeding companies, as described in Schulz et al. [17]. They were selected to cover a large variation for different phenotypic traits. All cultivars were grafted onto *R. corymbifera* ‘Laxa’ and cultivated in seven-liter pots using standard soil in a randomized block design with three blocks comprising one clone of each genotype in a greenhouse at the Plant Variety Office in Hannover, Germany. For the validation of the selected markers, we used an independent population of rose varieties grown in a field plot at the Federal Plant Variety Office in Hannover.

### 4.2. Phenotyping for Petal Number

For 95 genotypes of the association panel, the number of petals was counted for three flowers on each of three clones sampled at different time points during the season. For the 242 individuals of the validation population, the number of petals was counted for three flowers sampled at different time points during the season. Arithmetic means were calculated from the values for each genotype (Supplementary Figure S5).

### 4.3. Phenotyping for Petal Length

Petal length was measured in millimeters on graph paper as described above for petal number but only for 179 cultivars of the validation population (Supplementary Figure S6). It was calculated as the mean of the measurements of the five outermost petals from three flowers.

### 4.4. Phenotyping for Fragrance

Fragrance of 95 genotypes of the association panel was manually evaluated by three persons simply smelling the flowers. The fragrance was rated based on five classes: 0 = no fragrance, 1 = mild to moderate fragrance, 2 = good fragrance, 3 = strong fragrance, and 4 = very strong fragrance. The final median value was the result of the individual testing from the three persons. The fragrance data for the genotypes of the validation population were obtained from the data of the Federal Plant Variety Office in Hannover, which was rated based on three classes: 1 = no or low fragrance, 2 = medium fragrance, and 3 = strong fragrance.

### 4.5. DNA Extraction and Quantification

DNA was extracted using the NucleoSpin^®^ Plant II Kit (MACHEREY-NAGEL GmbH & Co. KG, Düren, Germany), according to the manufacturer protocol with small modifications: young, unfolded leaves were collected and stored for 24 h at room temperature in the dark. Fifty milligrams of frozen leaf tissue were homogenized in 2 mL test tubes with two steal beads using a bead mill (Retsch, Haan, Germany) at a frequency of 25 Hz for 2.5 min.

Using a Nanodrop 2000c spectrophotometer (PeQLab Biotechnologie GmbH, Erlangen, Germany), 1 µL of each DNA sample was quantified. The integrity of the DNA was evaluated by agarose gel analysis in comparison with λ-DNA standards (10 to 100 ng/μL) on 1% gels. Gels were run for 1 h at 100 V/cm, and the DNA concentrations of the samples were calculated with Gel-Pro Analyzer Software (Media Cybernetics, Rockville, MD, USA).

### 4.6. GWAS Analysis

The association panel of 95 individuals was designed to reduce the genetic relatedness between genotypes. The values for the nonnormally distributed traits, petal number and fragrance, were Box-Cox transformed before GWAS analysis. The GWAS analyses were performed using the mixed linear model (MLM, K + Q) in Tassel 3.0 [20]. A total of 63,000 SNP markers from the WagRhSNP Axiom array [16] with a minor allele frequency of more than 0.05 were included in the analysis. SNP genotypes were reduced to a diploid configuration for analysis in TASSEL 3.0. All heterozygous loci were encoded as AB, and homozygous loci were encoded as either AA or BB [16]. Significance thresholds were corrected for multiple testing by using the Bonferroni method using the number of contigs (28,054) to a significance threshold of 1.78 × 10^−6^. The population structure was calculated with the software STRUCTURE 2.3.4, and the kinship matrix with the software SPAGeDi 1.5 as described in Schulz et al. [17]. The Linkage Disequilibrium (LD) among SNP markers across the genome was estimated in TASSEL 3 and the output was used to construct a LD decay plot using the software R (Supplementary Figure S7).

### 4.7. KASP-Assay for SNP Validation

SNP markers for Kompetitive Alelle Specific PCR (KASP) assays were designed by LGC Genomics (London, UK). Genotyping was performed using a StepOnePlus or Quantstudio 6 Real-Time PCR system (Applied Biosystems, Foster City, CA, USA) with 20 ng DNA, 5 µL KASP V4.0 Mastermix 96/384, High Rox, 0.14 µL KASP by Design Primer Mix in a final volume of 10 µL for each reaction. KASP thermocycling was performed according to the manufacturer’s standard protocol: activation for 15 min at 94 °C, followed by 10 cycles at 94 °C for 20 s and 61 °C for 1 min (61 °C decreasing 0.6 °C per cycle to achieve a final annealing temperature of 55 °C); followed by 26 cycles at 94 °C for 20 s and 55 °C for 1 min. Reading of KASP genotyping reactions on the qPCR machine was performed in a final cycle at 30 °C for 30 s. If fluorescence data did not form satisfying clusters, the conditions for additional cycles were 94 °C for 20 s, followed by 57 °C for 1 min (up to 3 cycles). Genotypic data were analyzed with QuantStudio Software v3.1 (Applied Biosystems, Waltham, MA, USA).

### 4.8. Statistical Analysis

The nonparametric Kruskal–Wallis rank-sum test was used to identify significant differences in the mean SNP effect in groups of cultivars for the petal number and fragrance traits (*p* < 0.05; Dunn’s method). To identify significant differences in petal length, all pairwise multiple comparison procedures were used (overall significance level = 0.05; Holm-Sidak method).

Phenotypic data were tested for normal distribution using the Shapiro-Wilk test (α = 0.05). The data that were not normally distributed were transformed using the Box-Cox transformation [65]. The statistical calculations were performed in Excel 2016, MYSTAT 12 (Systat Software, Inc., San Jose, CA, USA), Past 4.02 [66] and QtiPlot 1.0.0. The Manhattan plots were constructed with the package CMplot in the software R 3.4.1 using the output from Tassel 3.0.

## 5. Conclusions

Petal number, petal length and floral fragrance in roses are genetically complex traits, with each having a number of loci influencing trait expression. However, among these factors, we identified major QTL for each trait, and for each trait, we also established KASP markers that were validated in independent populations of garden roses. Although each marker explains only part of the genetic variation, it displays significant effects for each of the traits. Therefore, they are ideally suited as tools in breeding programs for improving the underlying traits directly or to increase the frequency of favorable alleles in the breeding pool, thereby improving the selection of trait combinations in the progeny. Furthermore, they might be starting points for positional cloning of the genes underlying the QTL.

## Data Availability

Original data is available upon request from the corresponding author.

## Supplementary Materials

The following are available online at https://www.mdpi.com/article/10.3390/plants10050897/s1, **Figure S1:** Skewed distribution of 223 cultivars for the validation of petal number and normal distribution of petal length in the validation population for 179 cultivars. **Figure S2:** Manhattan plot of associations between 63,000 SNP-markers and petal number in a set of 95 garden roses. **Figure S3:** Manhattan plot of associations between 63,000 SNP-markers and fragrance in of a set of 95 garden roses. **Figure S4:** Distribution of fragrance in the population for validation with 222 cultivars for KASP marker Rh_FR_SNP139K (a) and with 282 cultivars for KASP marker Rh_FR_SNP201K (b). **Figure S5****:** Examples for single flowered roses and very full flowered roses. **Figure S6:** Examples for roses with long petals and very short petals. **Figure S7:** Genome wide LD decay plot. **Table S1:** Distribution of allele dosage of KASP marker Rh_PN_SNP5K for petal number in the association panel of 95 cultivars. **Table S2:** Validation of KASP marker Rh_PN_SNP5K for petal number with 223 independent rose cultivars. **Table S3:** Distribution of allele dosage of KASP marker Rh_PL_SNP49K for petal length in the association panel of 95 cultivars. **Table S4:** Validation of KASP marker Rh_PL_SNP49K for petal length with 179 independent rose cultivars. **Table S5:** Distribution of allele dosage of KASP marker Rh_FR_SNP139K for fragrance in the association panel of 95 cultivars. **Table S6:** Validation of KASP marker Rh_FR_SNP139K with 222 independent rose cultivars. **Table S7:** Distribution of allele dosage of KASP marker Rh_FR_SNP201K for fragrance in the association panel of 95 cultivars. **Table S8:** Validation of KASP marker Rh_FR_SNP201K with 282 independent rose cultivars. **Table S9:** Significant SNPs associated with petal number in roses. **Table S10:** Significant SNPs associated with petal length in roses. **Table S11:** Significant SNPs associated with fragrance in roses.
