# Supplementary material for: Detection of Reproducible Major Effect QTL for Petal Traits in Garden Roses
Source: Plants (Basel). 2021 Apr 29;10(5):897. doi: 10.3390/plants10050897 (PMC8145204; doi:10.3390/plants10050897)
Supplement: Supplementary file 1 [file plants-10-00897-s001.zip › plants-1178715-supplementary.pdf]

FIGURES

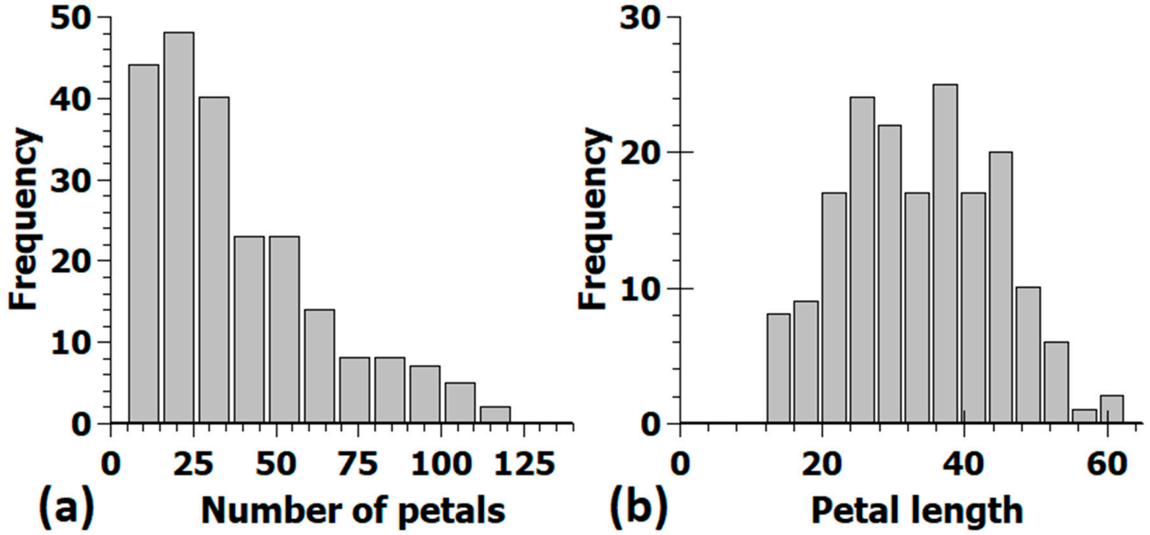

Suppl. Figure S1. Skewed distribution of 223 cultivars for the validation of petal number and normal distribution of petal length in the validation population for 179 cultivars (b).

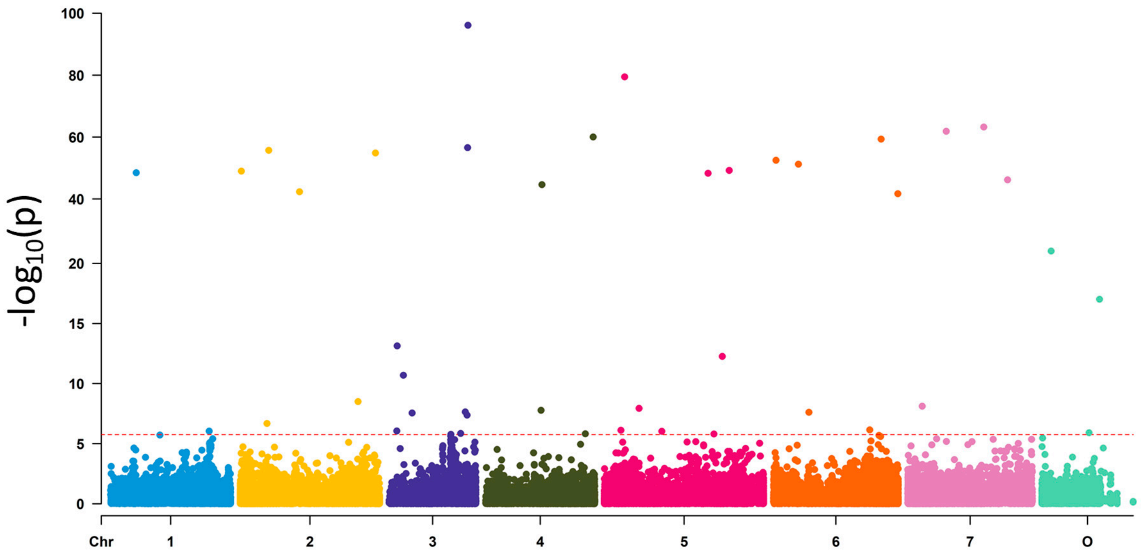

Suppl. Figure S2. Manhattan plot of associations between 63,000 SNP-markers and petal number in a set of 95 garden roses. The x-axis represents the chromosomes of *Rosa chinensis*, including chromosome 0 with contigs not assigned to a precise location to date. Associations were calculated in Tassel 3.0 using MLM including population structure (Q) and kinship (K) and Box-Cox transformed data for petal number. Red dotted line indicates the Bonferroni corrected level of significance (-log10 = 5.75).

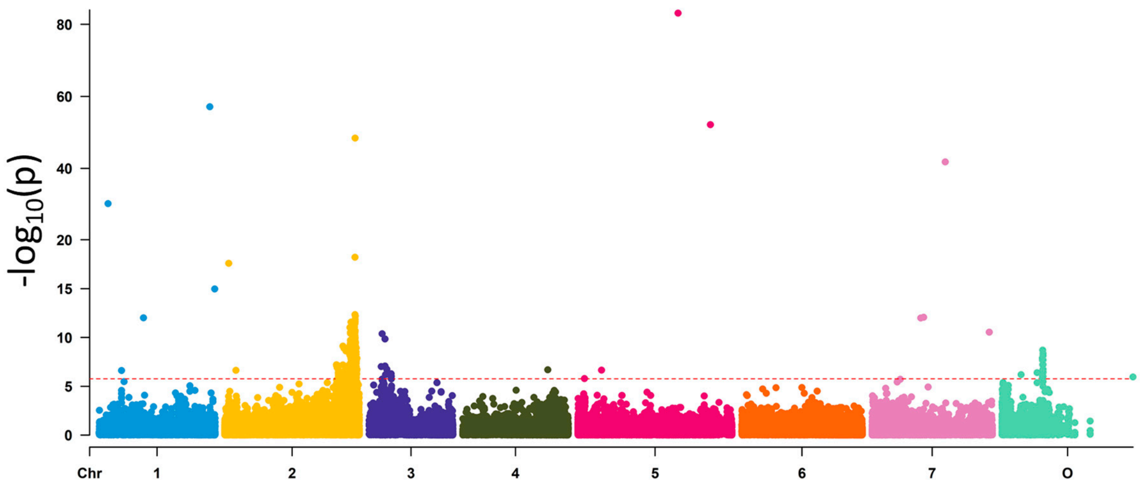

Suppl. Figure S3. Manhattan plot of associations between 63,000 SNP-markers and fragrance in of a set of 95 garden roses. The x-axis represents the chromosomes of *Rosa chinensis*, including chromosome 0 with contigs not assigned to a precise location to date. Associations were calculated in Tassel 3.0 using MLM including population structure (Q) and kinship (K) and Box-Cox transformed data of fragrance. Red dotted line indicates the Bonferroni corrected level of significance (-log10= 5.75).

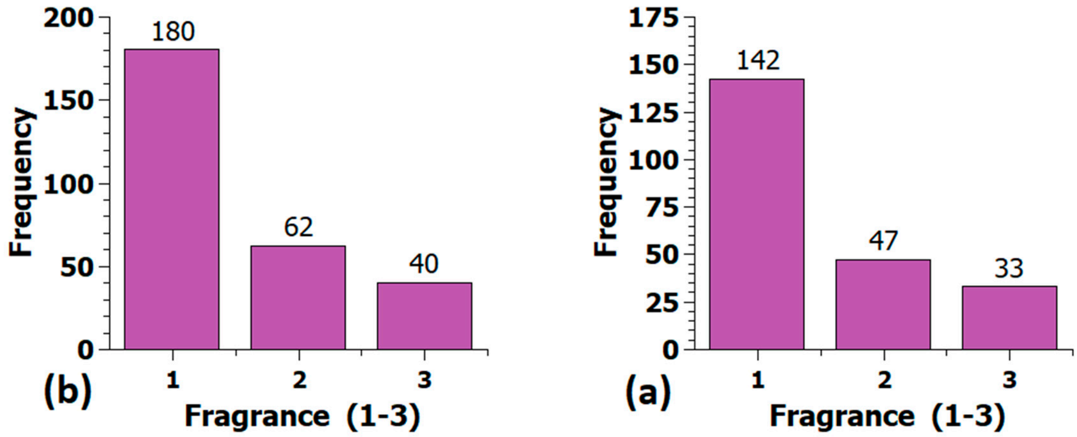

Suppl. Figure S4. Distribution of fragrance in the population for validation with 222 cultivars for KASP marker Rh_FR_SNP139K (a) and with 282 cultivars for KASP marker Rh_FR_SNP201K (b).

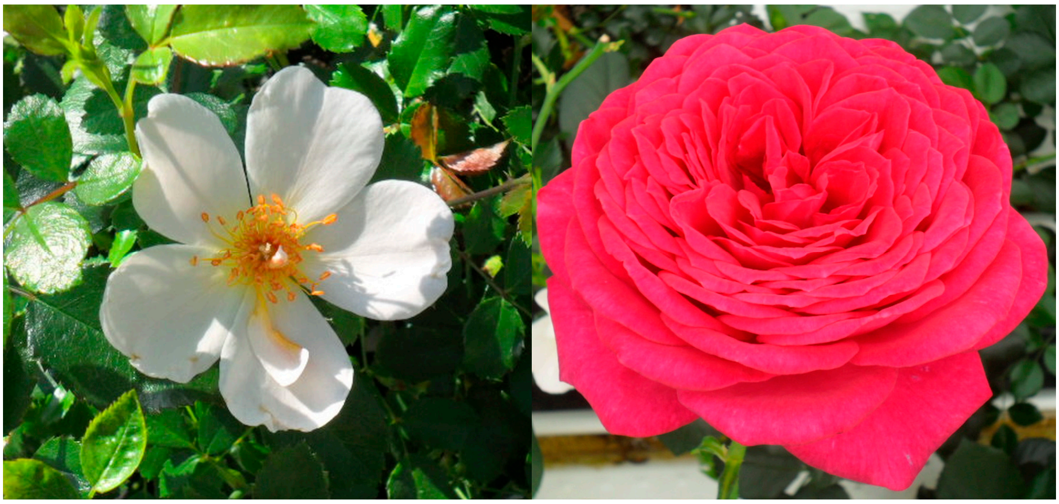

Suppl. Figure S5. Examples for single flowered roses (left; cultivar `Cute Haze´ with 6 petals) and very full flowered roses (right; cultivar `Goethe Rose´ with more than 100 petals).

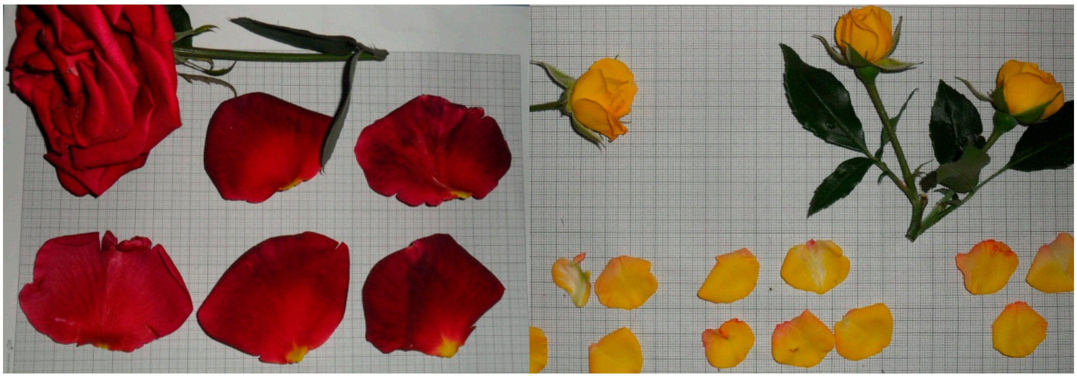

Suppl. Figure S6. Examples for roses with long petals (left; cultivar `Mister Lincoln´ with a petal length > 60mm) and very short petals (right; cultivar `Rumba´ with a petal length < 25 mm).

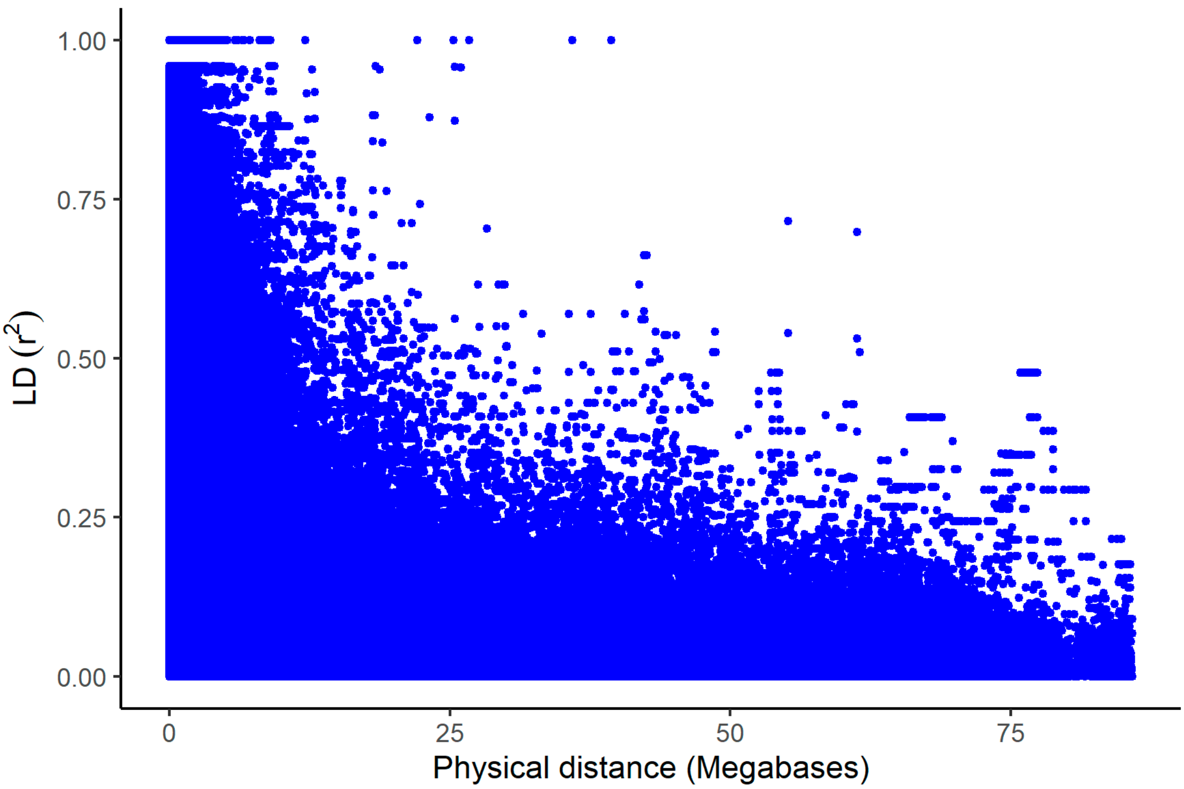

Suppl. Figure S7. Genome wide LD decay plot.

TABLES

Suppl. Table S1. Distribution of allele dosage of KASP marker Rh_PN_SNP5K for petal number in the association panel of 95 cultivars.

No.	Cultivar	Petals (n)	SNP Dosage	No.	Cultivar	Petals (n)	SNP Dosage
134	Albrecht Dürer Rose	82.32	0	72	Arabia	28.22	1
37	Alinka	31.52	0	44	Arthur Bell	40.08	1
38	Auslo	85.42	0	55	Ausfather	61.18	1
104	Beverly	55.04	0	39	Ausmas	51.53	1
136	Bienenweide	7.52	0	85	Berolina	38.83	1
53	Celine Delbard	29.62	0	36	Black Baccara	45.81	1
49	Compassion	39.02	0	66	Black Magic	52.65	1
46	Comtesse de Ségur	31.5	0	67	China Girl	77.72	1
140	Crimson Glory	31.7	0	10	Chippendale	119.38	1
95	Dortmund	7.25	0	69	Comtessa AL	95.74	1
132	Duftwolke	41.07	0	131	Cute Haze	7.87	1
115	Focus	32.34	0	111	Donauprinzessin	69.32	1
92	Frühlingsduft	23	0	75	Elfe	79.67	1
15	George Vancouver	23.28	0	13	Feuerwerk	16	1
133	Goethe Rose	107.17	0	59	France Libre	24.65	1
24	Heidetraum	27.48	0	96	Friesia	37.24	1
100	Herkules	69.3	0	103	Fritz Nobis	20.67	1
120	Herzogin Friederike	30.17	0	14	Gebrüder Grimm	43.07	1
18	Jasmina	65.07	0	73	Hansestadt Rostock	26.99	1
105	Juanita	5.07	0	17	Heidi Klum	96.42	1
16	König Stanislaus	30.01	0	77	Jazz	15.57	1
118	Kronjuwel	19.93	0	74	Kastelruther Spatzen	76.44	1
41	La Sevillana	19.6	0	84	Knock Out	12.64	1
70	Lipstick	23.63	0	94	Lavender Lassie	51.6	1
71	Midsummer	53.89	0	137	Lolita	48.24	1
42	Mister Lincoln	33.5	0	54	Louise Odier	75.68	1
47	Mme Boll	91.11	0	138	Magenta	78.6	1
57	Mme Knorr	110.78	0	81	Mariatheresia	128.67	1
62	Mrs John Laing	60.11	0	32	Mevrouv Nathal. Nypels	80.78	1
112	Münsterland	30	0	35	Mitsouko	71.6	1
4	Nemo	5.83	0	80	My Girl	115.38	1
28	New Dawn	41.63	0	58	Papageno	31.17	1
26	Nostalgie	38.02	0	56	Perpetually Yours	197	1
1	Parole	37.57	0	52	Rose de Resht	126.55	1
68	Perennial Blush	12.66	0	139	Rose Gaujard	54.21	1
2	Queen Elizabeth	31.51	0	43	Rumba	33.76	1
99	Raubritter	24.47	0	3	Schneewittchen	28.05	1
51	Scarlet Meidiland	18.88	0	93	Sebastian Kneipp	108.5	1
40	Shalom	51.06	0	141	Sunset Boulevard	22	1
116	Simply	17.2	0	119	Tornella	33.06	1
27	Sommerwind	19.6	0	12	Blue Parfum	51.45	2
20	Sonnenschirm	38.22	0	122	Blue River	45.9	2
135	Stadt Rom	5.8	0	79	Dukat	24	2
5	Super Star	36.72	0	78	Mainzer Fastnacht	41.5	2
50	Sutters Gold	19.92	0	61	Princess Alexandre	52.74	2
114	Venice	5.46	0	6	Small Maidens Blush	128.77	2
89	Westerland	23.5	0	97	Sterntaler	79.75	2
110	Windrose	16.33	0				

Suppl. Table S2. Validation of KASP marker Rh_PN_SNP5K for petal number with 223 independent rose cultivars.

Cultivar	SNP dosage	Petal (n)	Cultivar	SNP dosage	Petals (n)	Cultivar	SNP dosage	Petals (n)
Alden Biesen	0	6	Pastella	0	66	Gartenspaß	1	57.5
Alpenglühen	0	9.33	Perennial Blue	0	30.5	Gentile Harmonie	1	67.33
Anda	0	6	Petticoat	0	30.33	Goldelse	1	48
Annelies	0	46	Piano	0	95.5	Golden Monica	1	23
Antique Silk	0	30.33	Pink Bassino	0	7	Golden Silk	1	28.67
Apache	0	5.25	Planten un Blomen	0	32	Golden Wings	1	5
Ascot	0	63.25	Pomponella	0	54.2	Goldmarie 82	1	23.44
Aspirin Rose	0	32.53	Pretty Pink	0	5	Goldschatz	1	32
Astrid Gräfin. von Hardenberg	0	71	Princesse Josephine - Charlotte	0	57.4	Grete Schichedanz	1	38.33
Bad Wörishofen 2005	0	21	Puccini	0	5.67	Helene de Gerlach	1	24.5
Barkarole	0	35	Purple Kiss	0	11.33	Hella	1	19
Bel Esprit	0	5	Purple Roadrunner	0	60	Inka	1	90
Bienenweide apricot	0	6.17	Ravel	0	5	Juwena	1	42.67
Bienenweide gelb	0	23.55	Red Haze	0	6	Lady Rose	1	21.5
Bienenweide rot	0	5	Robusta	0	5	Ladybird	1	41
Blühwunder 08	0	15.67	Rodeo	0	14.67	Lampion	1	49.44
Brigitte de Villenjagne	0	30.2	Rody	0	33.67	Liebeszauber	1	35.5
Camelot	0	39.25	Rokoko	0	21.5	Maria Teresa	1	24.5
Capricia	0	32.5	Romanze	0	16.2	Maxim	1	55.22
Catherine Laborde	0	31.33	Rosanna	0	57.5	Meimelliac	1	65
Cherry Girl	0	31.5	Rosario	0	30	Memoire	1	50
Christel von der Post	0	33	Rosarium Uetersen	0	85	Mondial	1	39.5
Claire Jolly	0	12	Rose von Oldenburg	0	47.5	Morning Sun	1	29
Delicia	0	22	Rosenprof. Sieber	0	23.33	Neige d'Ete	1	24
Dinky	0	35	Rosenstadt Freising	0	30.75	Omi Oswald	1	9.5
Easy to cut	0	22.33	Rosy Purple	0	5	Osiana	1	25
Eifelzauber	0	71	Roter Korsar	0	15.5	Pearl Mirato	1	32
Elvis	0	46.33	Rotilia	0	22	Pink Roadrunner	1	25
Emil Nolde Rose	0	30.67	Running Maid	0	5.33	Postillion	1	31.67
Escimo	0	5	Sana Rose	0	43.9	Racquel	1	30
Fairest Cape	0	24.4	Satina	0	22.67	Rebell	1	25
Feeling	0	18.67	Schloß Ippenburg	0	13	Romina	1	82
Felicita	0	43.33	Shanty	0	24	Rose de la Petit Chabote	1	33.5
Felicitas	0	5	Sibelius	0	20	Rosenfee	1	50
Fils de Saison	0	6.67	Sommerabend	0	5.75	Rosengrf. Marie Antoinette	1	79.33
Flash	0	16	Sonnenröschen	0	5	Rugelda	1	34
Fortissima	0	5	Souvenir de Baden Baden	0	49	Scepter´s Isle	1	49.2
Fortuna	0	5.33	Sunny Rose	0	22.67	Schwarze Madonna	1	33
Francoise Drion	0	5	Sunny Sky	0	44.25	Sirius	1	34.33
Garden of Roses	0	95	The Mayflower	0	96.33	Solero	1	74.5
Gelber Engel	0	17.33	Tornado	0	18.3	Sommersonne	1	44.5
Giardinia	0	94.4	Ueters. Rosenkönigin	0	112	Soul	1	95
Golden Tower	0	51	Uetersener Rosen-prinzessin	0	103.5	Strawberry	1	88
Hans Gönewein Rose	0	27	Vodacom	0	53.33	Summer Memories	1	72.5
Hansaland	0	25.17	Vogelpark Walsrode	0	12.5	T06622	1	90.2
Harlow Carr	0	121.67	Waterloo	0	53.33	Uetersen. Klosterrose	1	45.38
Heavenly Pink	0	25	White Chrystal	0	5	Ulmer Münster	1	31
Home and Garden	0	55	White Haze	0	7.67	Valencia	1	50
Internescori	0	58.3	White Magic	0	5.4	Vanilla Cream	1	23.33
Jet Spray	0	20	Yellow light	0	15	Vif Eclat	1	5
Jour de Fete	0	25	Zwergfee 09	0	27.5	Walzertraum	1	56.33
Kosmos	0	68.67	Acapella	1	58.5	Zaide	1	132.5

La Perla	0	64	Altera	1	35.2	Aperitif	2	31
Larissa	0	106	Amber Sun	1	16	Avec Amour	2	31.4
Lilly Marleen	0	18	Aphrodite	1	42.83	Bouquet Parfait	2	37.5
Lions Rose	0	45	Aprikola	1	23.67	Candela	2	53.67
Lupo	0	6	Artemis	1	85	Candlelight	2	41.5
Maaseik	0	6	Augusta Luise	1	55	Cubana	2	26
Mainaufeuer	0	22.33	Aya Renaissance	1	59.33	Eskelund	2	56.33
Maria Mathilda	0	17.67	Bad Birnbach	1	28.67	Golden Medaillon	2	31.5
Marie-Luise Marjan	0	30	Belvedere	1	15.8	Jaqueline Humery	2	22.33
Matador	0	12.5	Bernstein-Rose	1	35	Karl Ploberger	2	87
Matchball	0	5	Blanche Moreau	1	71	Madame Bovary	2	29.5
Medeo	0	9.3	Bremer Stadt-musikanten	1	79	Parfum de Grasse	2	45.75
Mein schöner Garten	0	35	Bukavu Lenbrirus	1	5.67	Playful Rokoko	2	64.33
Melina	0	108	Canary	1	46	Voyage	2	109
Mirato	0	23	Caramella	1	40	Alabaster	3	81.67
Mon Petit Chou	0	77.5	Centro Rose	1	20	Apricot Clementine	3	17.33
Moneta	0	40.67	Claus Dalby	1	87.76	Brokat - Tantakorb	3	40
Mozart	0	5	Deep Impression	1	50	Freisinger Morgenröte	3	25
Nathalie Renaissance	0	105	Domstadt Fulda	1	60	Ninetta	3	66.8
NDR1 Radio Niedersachsen	0	22.67	Elmshorn	1	52.67	Queen of Heart	3	89
Palmengarten Frankfurt	0	22.5	Fantasia Mondiale	1	29.67	Speelwark	3	42
Paprika	0	20.6	First Lady	1	62.5			
Pascaline	0	41	Frisson Frais	1	5			

Suppl. Table S3. Distribution of allele dosage of KASP marker Rh_PL_SNP49K for petal length in the association panel of 95 cultivars.

No.	Cultivar	SNP dosage	Petal length (mm)	No.	Cultivar	SNP dosage	Petal length (mm)
1	Parole	3	65.39	69	Comtessa AL	1	50.91
2	Queen Elisabeth	1	57.7	70	Lipstick	0	25.05
3	Schneewittchen	2	40.23	71	Midsummer	0	47.66
4	Nemo	0	24.99	72	Arabia	1	46.26
5	Super Star	2	52.56	73	Hansestadt Rostock	1	48.72
6	Small Maidens Blush	0	34.01	74	Kastelruther Spatzen	0	30.61
10	Chippendale	3	41.49	75	Elfe	2	68.2
12	Blue Parfum	1	46.67	77	Jazz	0	33.87
13	Feuerwerk	1	50.37	78	Mainzer Fastnacht	1	53.85
14	Gebrüder Grimm	1	42.51	79	Dukat	1	52.85
15	George Vancouver	1	34.73	80	My Girl	2	50.06
16	König Stanislaus	0	46.37	81	Mariatheresia	0	34.99
17	Heidi Klum	1	37.5	84	Knockout	1	47.74
18	Jasmina	0	33.21	85	Berolina	1	67.7
20	Sonnenschirm	2	36.23	89	Westerland	1	60.73
24	Heidetraum	0	29.67	92	Frühlingsduft	1	54.47
26	Nostalgie	2	38.88	93	Sebastian Kneipp	0	52.8
27	Sommerwind	0	34.3	94	Lavender Lassie	3	37.24
28	New Dawn	0	42.66	95	Dortmund	1	56.55
32	Mevrouv N. Nypels	0	42.23	96	Friesia	4	53
35	Mitsouko	1	54.69	97	Sterntaler	1	47.1
36	Black Baccara	2	45.16	99	Raubritter	0	34.02
37	Alinka	1	48.99	100	Herkules	1	46.83
38	Auslo	1	48.35	103	Fritz Nobis	1	40.62
39	Ausmas	1	58.18	104	Beverly	0	55.74
40	Shalom	1	46.16	105	Juanita	0	21.61
41	La Sevillana	0	36.57	110	Windrose	2	42.5
42	Mister Lincoln	2	64.12	111	Donauprinzessin	0	37.21
43	Rumba	0	22.95	112	Münsterland	2	60.93

44	Arthur Bell	1	66.29	114	Venice	0	34.93
46	Comtesse de Segur	2	53.1	115	Focus	1	57.16
47	Mme Boll	1	46.84	116	Simply	0	40.5
49	Compassion	1	59.76	118	Kronjuwel	1	33.85
50	Sutters Gold	2	66.5	119	Tornella	1	37.35
51	Scarlet Meidiland	0	22.8	120	Herzogin Friederike	0	32.73
52	Rose de Resht	0	32.43	122	Blue River	1	51.8
53	Celine Delbard	1	49.01	131	Cute Haze	0	28.28
54	Louise Odier	2	36.94	132	Duftwolke	1	54.35
55	Ausfather	1	52.38	133	Goethe Rose	3	61.35
56	Perpetually Yours	0	44.3	134	Albrecht Dürer Rose	2	41.5
57	Mme Knorr	1	46.38	135	Stadt Rom	0	29.77
58	Papageno	0	46.77	136	Bienenweide	0	24.93
59	France Libre	3	50.69	137	Lolita	2	54.54
61	Princess Alexandra	1	45.65	138	Magenta	2	38.81
62	Mrs John Laing	2	60.4	139	Rose Gaujard	3	50.88
66	Black Magic	2	50.82	140	Crimson Glory	1	55.61
67	China Girl	2	49.77	141	Sunset Boulevard	3	57.08
68	Perennial Blush	0	20.64				

Suppl. Table S4. Validation of KASP marker Rh_PL_SNP49K for petal length with 179 independent rose cultivars.

Cultivar	SNP dosage	Petal length (mm)	Cultivar	SNP dosage	Petal length
Alden Biesen	0	17.6	Arabia	1	38.0
Alpenglühen	0	20.8	Artemis	1	34.4
Annelies	0	17.7	Ascot	1	47.0
Apricot Clementine	0	23.3	Ausrimini Strawberry	1	41.7
Aspririn	0	27.9	Aya Renaissance	1	41.9
Bad Birnbach	0	22.9	Bienenweide, gelb	1	25.9
Bad Wörishofen	0	23.1	Bouquet Parfait	1	24.8
Barkarole	0	53.0	Brokat	1	45.4
Bel Esprit	0	23.5	Bukava	1	25.9
Bienenweide, apricot	0	22.2	Cap Diamant	1	35.9
Bienenweide, rot	0	25.0	Catherine Laborde	1	49.8
Blühwunder	0	21.9	Deep Impression	1	33.4
Brigitte de Villenfagne	0	16.3	Dukat	1	51.8
Caesera	0	37.0	Elvis	1	47.1
Candy	0	36.8	Eskelund	1	47.9
Capricia	0	34.8	Fairest Cape	1	39.4
Cherry Lips	0	15.5	Freisinger Morgenröte	1	45.0
Cinderella	0	27.0	Gartenspaß	1	33.0
Diamant	0	24.1	Goldelse	1	34.5
Eifelzauber	0	42.2	Golden Wings	1	42.3
Escimo	0	33.3	Harlow Carr	1	34.9
Fantasia Mondiale	0	45.3	Heavenly Pink	1	12.6
Felicita	0	32.1	Juwena	1	26.8
Felicitas	0	25.7	La Perla	1	41.3
Flash	0	15.1	Ladybird	1	35.9
Fortissima	0	18.9	Lampion	1	38.5
Fortuna	0	15.6	Lions Rose	1	38.1
Geisha	0	30.7	Mainaufeuer	1	28.4
Giardina	0	41.4	Maria Teresa	1	30.0
Goldmarie	0	29.0	Maxim	1	52.3
Goldschatz	0	27.2	Mein schöner Garten	1	34.4
Home and Garden	0	30.9	Natalie Renaissance	1	47.9
Internescori	0	40.3	Ninetta	1	27.2
Jacqueline Humery	0	25.2	Paprika	1	35.8
Jet Spray	0	12.0	Parfume de Grasse	1	41.1
Larissa	0	22.8	Purple Kiss	1	21.4
Liebeszauber	0	45.3	Rose de la Petite Chabot	1	26.7

Maria Mathilda	0	30.5	Rose von Oldenburg	1	28.3
Marie-Luise Marjahn	0	42.4	Rosengräfin Marie Henriette	1	40.9
Marvella	0	36.9	Rosenprofessor Sieber	1	31.1
Matador	0	25.7	Roter Korsar	1	38.2
Matchball	0	21.4	Sangerhausen	1	37.1
Medeo	0	15.9	Souvenir de Baden Baden	1	44.7
Melina	0	34.3	T10605 Maxim	1	52.3
Mirato	0	24.5	Zaide	1	44.4
Mondial	0	46.4	Acapella	2	62.0
Moneta	0	23.3	Aphrodite	2	62.7
Mozart	0	13.9	Avec Amour	2	57.5
NDR3	0	30.0	Bassino	2	16.5
Palmengarten frankfurt	0	24.1	Bernsteinrose	2	28.3
Pastella	0	27.7	Cherry Girl	2	27.7
Perennial Blue	0	14.9	Claus Dalby	2	46.9
Pink Bassino	0	25.6	Domstadt Fulda	2	30.1
Planten un Bloomen	0	26.5	Feeling	2	26.1
Playful Rokoko	0	22.9	Friedenslicht	2	36.4
Pomponella	0	29.2	Garden of Roses	2	35.0
Queen of Hearts	0	36.8	Gentile Harmonie	2	45.8
R. rugosa SchneeEule	0	45.1	Golden Monica	2	38.2
Racquel	0	48.4	Golden Silk	2	51.6
Red Haze	0	33.5	Helene de Gerlach	2	43.2
Rody	0	25.2	Inka	2	35.7
Romanze	0	38.1	Kosmos	2	34.1
Rotilia	0	26.5	Kupferkönigin	2	45.8
Sana Rose	0	34.0	Mme Bovary	2	46.5
Satina	0	15.9	Morning Sun	2	29.3
Scepter´s isle	0	44.8	Olympisches Feuer	2	25.0
Shirley	0	43.3	Omi Oswald	2	24.0
Sirius	0	39.8	Piano	2	30.7
Sommerabend	0	20.6	Postillon	2	47.3
Sonnenröschen	0	17.3	Purple Lodge	2	38.9
Soul	0	43.4	Rokoko	2	44.6
Souvenir	0	29.1	Romina	2	60.0
Stadt Rom	0	28.8	Schloß Ippenburg	2	50.9
T06622	0	39.4	Shanty	2	38.4
The Mayflower	0	41.1	Vanilla Cream	2	22.5
Tornado	0	31.5	Voyage	2	48.9
Uetersener Klosterrose	0	47.1	Amber Sun	3	30.2
Uetersener Rosenkönigin	0	33.3	Candlelight	3	42.2
Uetersener Rosenprinzessin	0	31.7	Caramella	3	37.7
Walzertraum	0	46.9	Gelber Engel	3	27.2
Waterloo	0	17.1	Golden Medaillon	3	43.3
White Chrystal	0	21.8	Grande Amore	3	52.0
White Haze	0	36.1	Lilly Marleen	3	35.7
White Magic	0	20.5	Meimelliac	3	47.3
Yellow Light	0	14.4	Robusta	3	36.5
Zwergenfee	0	22.6	Rugelda	3	37.4
Alabaster	1	29.7	Schwarze Madonna	3	39.3
Altera	1	42.8	Solero	3	26.5
Aperitif	1	38.3	Jubilé Imperial	4	38.4
Aprikola	1	30.5			

Suppl. Table S5. Distribution of allele dosage of KASP marker Rh_FR_SNP139K for fragrance in the association panel of 95 cultivars.

No.	Cultivar	SNP dosage	Scent	No.	Cultivar	SNP dosage	Scent
131	Cute Haze	0	0.63	80	My Girl	3	0.88
41	La Sevillana	0	1.16	58	Papageno	3	0.88
4	Nemo	0	0.21	2	Queen Elizabeth	3	0.75
51	Scarlet Meidiland	0	0.25	139	Rose Gaujard	3	1.3
135	Stadt Rom	0	0.3	3	Schneewittchen	3	1.13
37	Alinka	1	1	20	Sonnenschirm	3	1.17
136	Bienenweide	1	0.25	97	Sterntaler	3	2.12
15	George Vancouver	1	0.63	141	Sunset Boulevard	3	0.75
24	Heidetraum	1	0.88	5	Superstar	3	1.15
18	Jasmina	1	1.5	119	Tornella	3	0.75
74	Kastelruther Spatzen	1	0.72	89	Westerland	3	2.5
70	Lipstick	1	1.34	110	Windrose	3	1.04
116	Simply	1	1.11	72	Arabia	4	2
27	Sommerwind	1	1.05	55	Ausfather	4	2.75
36	Black Baccara	2	0.25	38	Auslo	4	3.25
66	Black Magic	2	1.88	39	Ausmas	4	1.69
46	Comtesse de Segur	2	2.5	104	Beverly	4	3
111	Donauprinzessin	2	1.25	12	Blue Parfum	4	3.5
59	France Libre	2	0.5	122	Blue River	4	3.5
14	Gebrüder Grimm	2	1.25	49	Compassion	4	3
73	Hansestadt Rostock	2	0.75	69	Comtessa AL	4	2.75
77	Jazz	2	1.25	140	Crimson Glory	4	3
105	Juanita	2	0.75	132	Duftwolke	4	3.5
118	Kronjuwel	2	1.06	96	Friesia	4	2.63
81	Mariatheresia	2	1.23	103	Fritz Nobis	4	3.25
32	Mevrouv N. Nypels	2	1.03	92	Frühlingsduft	4	2.5
71	Midsummer	2	0.46	133	Goethe Rose	4	3
28	New Dawn	2	1.17	17	Heidi Klum	4	2.38
26	Nostalgie	2	0.82	100	Herkules	4	3
99	Raubritter	2	2.75	94	Lavender Lassie	4	3.13
43	Rumba	2	1.07	54	Louise Odier	4	3
40	Shalom	2	0.75	138	Magenta	4	3.21
114	Venice	2	1.09	78	Mainzer Fastnacht	4	3.4
134	A. Dürer Rose	3	1.75	42	Mister Lincoln	4	2.63
44	Arthur Bell	3	1.25	35	Mitsouko	4	1.72
85	Berolina	3	2	57	Mme Knorr	4	2.75
53	Celine Delbard	3	1	47	Mme. Boll	4	2.92
67	China Girl	3	1.25	62	Mrs. John Laing	4	2.94
10	Chippendale	3	2	112	Münsterland	4	2.06
95	Dortmund	3	0.5	1	Parole	4	2.5
79	Dukat	3	2.5	68	Perennial Blush	4	3
75	Elfe	3	1	56	Perpetually Yours	4	0.25
13	Feuerwerk	3	1.5	61	Princess Alexandra	4	3.19
115	Focus	3	1.63	52	Rose de Resht	4	3.07
120	Herzogin Friederike	3	0.5	93	Sebastian Kneipp	4	2.75
84	Knockout	3	0.96	6	Small Maidens	4	2.75
16	König Stanislaus	3	1.63	50	Sutters Gold	4	3.5
137	Lolita	3	2.38				

Suppl. Table S6. Validation of KASP marker Rh_FR_SNP139K with 222 independent rose cultivars

Cultivar	Scent	SNP dosage	Cultivar	Scent	SNP dosage	Cultivar	Scent	SNP dosage
Satina	1	0	Bienenweide apricot	1	2	Zaide	1	4
Rody	1	0	Apricot Clementine	1	2	White Chrystal	2	4
Red Haze	1	0	Annelies	1	2	Vanilla Cream	1	4
Purple Kiss	1	0	Alden Biesen	1	2	Souvenier de Rose Marie	1	4
Pearl Mirato	1	0	Unicef Rose	1	2	Solero	1	4
Mirato	1	0	Giardina	2	2	Schoone Gezelle Blomme	1	4
Medeo	1	0	Perennial Blue	3	2	Sana Rose	1	4
Fortuna	1	0	Maxim	3	2	Rugelda	1	4
Centro-Rose	1	0	Jour de Fetes	3	2	Rosy Purple	1	4
Amulett	1	0	Walferdange	1	3	Pretty Pink	1	4
Alpenglühen	1	0	Ueters. Rosenprinzessin	1	3	Poesie	1	4
White Magic	1	1	Ueters.Rosenkönigin	1	3	Omi Oswald	1	4
White Haze	1	1	Sunny Rose	1	3	Neon	1	4
Sommersonne	1	1	Sangerhausen Jubiläumsrose	1	3	NDR1 Radio Niedersachsen	1	4
Smile	1	1	Shanty	1	3	Matchball	1	4
Roter Korsar	1	1	Sonnenröschen	1	3	Maaseik	1	4
Purple Haze	1	1	Sahara	1	3	La Perla	1	4
Princ. J.-Charlotte	1	1	Rosenfee	1	3	Juwena	1	4
Playful Rokoko	1	1	Rose de la Petit Chabote	1	3	Helene de Gerlach	1	4
Planten un Blomen	1	1	Queen of Heart	1	3	Golden Wings	1	4
Pink Bassino	1	1	Osiana	1	3	Gelber Engel	1	4
Pastella	1	1	Morning sun	1	3	Fortissima	1	4
Paprika	1	1	Mein schöner Garten	1	3	Eliza	1	4
Palmengarten Frankfurt	1	1	Marvelle	1	3	Christel von der Post	1	4
Ninetta	1	1	Maria Teresa	1	3	Caramella	1	4
Neige d'Ete	1	1	Maria Mathilda	1	3	Bienenweide gelb	1	4
Moneta	1	1	Lampion	1	3	Feeling	1	4
Matador	1	1	Jaqueline Humery	1	3	Yellow light	1	4
Mainaufeuer	1	1	Heavenly Pink	1	3	White Roadrunner	2	4
Magma	1	1	Goldschatz	1	3	Waterloo	2	4
Home and Garden	1	1	Golden Monica	1	3	Verdi	2	4
History	1	1	Friedenslicht	1	3	Twins	2	4
Grande Amore	1	1	First Lady	1	3	Shirley	2	4
Goldmarie 82	1	1	Felicitas	1	3	Pascaline	2	4
Fantasia Mondiale	1	1	Escimo	1	3	Marie-Luise Marjan	2	4
Dinky	1	1	Eifelzauber	1	3	Golden Silk	2	4
Cubana	1	1	Easy to cut	1	3	Elvis	3	4
Cherry Lips	1	1	Dornröschenschloss Sababurg	1	3	Duftzauber 84	2	4
Bel Esprit	1	1	Domstadt Fulda	1	3	Claus Dalby	2	4
Austriana	1	1	Candela	1	3	Catherine Laborde	2	4
Aspirin Rose	1	1	Bouquet Parfait	1	3	Caesera	2	4
Arifa	1	1	Avec Amour	1	3	Brigitte de Villenfagne	2	4
Kordes Brillant	1	1	Aprikola	1	3	Bernstein-Rose	2	4
Neue Revue	1	1	Amber Sun	1	3	Augusta Luise	3	4
Romanze	2	1	Goldesel	1	3	Frühlingsgold	2	4
Zwergfee 09	1	2	Kleopatra	1	3	Königin von Dänemark	2	4
Vif Eclat	1	2	Walzertraum	2	3	Lady Like	2	4
Tornado	1	2	Voyage	2	3	Maiden's Blush	2	4
T06622	1	2	Souvenir de Baden Baden	2	3	Tapis Volant	2	4
Sirius	1	2	Rosenstadt Freising	2	3	The Pilgrims	2	4
Silver River	1	2	Petticoat	2	3	The Mayflower	3	4
Sibelius	1	2	Parfum de Grasse	2	3	Soul	3	4
Rosenprofessor Sieber	1	2	Memoire	2	3	Shocking Blue	3	4
Rose von Oldenburg	1	2	Melina	2	3	Schloß Ippenburg	3	4
Rebell	1	2	Marie Antoinette	2	3	Scepter´s Isle	3	4
Puccini	1	2	Liebeszauber	2	3	Rosengräfin M. Henriette	3	4
Piano	1	2	La Paloma 85	2	3	Racquel	3	4

Paganini	1	2	Kupferkönigin	2	3	Purple Lodge	3	4
Mariandel	1	2	Inka	2	3	Pink Roadrunner	3	4
Lilly Marleen	1	2	Grete Schichedanz	2	3	Nat. Renaissance	3	4
Ladybird	1	2	Emil Nolde Rose	2	3	Meimelliac	3	4
Kahlua	1	2	Candlelight	2	3	Madame Bovary	3	4
Jugenliebe	1	2	Canary	2	3	Internescori	3	4
Jet Spray	1	2	Aya Renaissance	2	3	Harlow Carr	3	4
Helmut Kohl Rose	1	2	Ascot	2	3	Gentile Harmonie	3	4
Gartenspaß	1	2	Aperitif	2	3	Felicita	3	4
Frisson Frais	1	2	Dukat	2	3	Eskelund	3	4
Francoise Drion	1	2	Esmeralda	2	3	Duftrausch	3	4
Fairest Cape	1	2	Freisinger Morgenröte	2	3	Capricia	3	4
Diamant Rose	1	2	Lapponia	2	3	Cap Diamant	3	4
Deep Impression	1	2	Uetersener Klosterrose	3	3	Biedermeier Garden	3	4
Claire Jolly	1	2	Marc Chagall	3	3	Astrid Grf. v. Hardenberg	3	4
Cherry Girl	1	2	Artemis	3	3	Altera	3	4
Black Magic	1	2	Aphrodite	3	3	Acapella	3	4

Suppl. Table S7. Distribution of allele dosage of KASP marker Rh_FR_SNP201K for fragrance in the association panel of 95 cultivars.

No.	Cultivar	SNP Dosage	Scent	No.	Cultivar	SNP Dosage	Scent
6	Small Maidens	0	2.75	46	Comtesse de Segur	2	2.5
12	Blue Parfum	0	3.5	54	Louise Odier	2	3
17	Heidi Klum	0	2.38	55	Ausfather	2	2.75
49	Compassion	0	3	58	Papageno	2	0.88
61	Princess Alexandra	0	3.19	67	China Girl	2	1.25
68	Perennial Blush	0	3	72	Arabia	2	2
78	Mainzer Fastnacht	0	3.4	73	Hansestadt Rostock	2	0.75
96	Friesia	0	2.63	74	Kastelruther Spatzen	2	0.72
100	Herkules	0	3	77	Jazz	2	1.25
122	Blue River	0	3.5	89	Westerland	2	2.5
138	Magenta	0	3.21	92	Frühlingsduft	2	2.5
10	Chippendale	1	2	95	Dortmund	2	0.5
13	Feuerwerk	1	1.5	97	Sterntaler	2	2.12
14	Gebrüder Grimm	1	1.25	103	Fritz Nobis	2	3.25
18	Jasmina	1	1.5	105	Juanita	2	0.75
20	Sonnenschirm	1	1.17	111	Donauprinzessin	2	1.25
26	Nostalgie	1	0.82	118	Kronjuwel	2	1.06
27	Sommerwind	1	1.05	119	Tornella	2	0.75
28	New Dawn	1	1.17	120	Herzogin Friederike	2	0.5
36	Black Baccara	1	0.25	135	Stadt Rom	2	0.3
40	Shalom	1	0.75	136	Bienenweide	2	0.25
47	Mme. Boll	1	2.92	139	Rose Gaujard	2	1.3
52	Rose de Resht	1	3.07	140	Crimson Glory	2	3
56	Perpetually Yours	1	0.25	2	Queen Elizabeth	3	0.75
57	Mme Knorr	1	2.75	3	Schneewittchen	3	1.13
62	Mrs. John Laing	1	2.94	15	George Vancouver	3	0.63
66	Black Magic	1	1.88	16	König Stanislaus	3	1.63
69	Comtessa AL	1	2.75	24	Heidetraum	3	0.88
70	Lipstick	1	1.34	35	Mitsouko	3	1.72
75	Elfe	1	1	37	Alinka	3	1
79	Dukat	1	2.5	42	Mister Lincoln	3	2.63
80	My Girl	1	0.88	44	Arthur Bell	3	1.25
84	Knockout	1	0.96	50	Sutters Gold	3	3.5
93	Sebastian Kneipp	1	2.75	51	Scarlet Meidiland	3	0.25
99	Raubritter	1	2.75	53	Celine Delbard	3	1
104	Beverly	1	3	59	France Libre	3	0.5
112	Münsterland	1	2.06	71	Midsummer	3	0.46
132	Duftwolke	1	3.5	81	Mariatheresia	3	1.23

133	Goethe Rose	1	3	85	Berolina	3	2
134	Albrecht Dürer Rose	1	1.75	94	Lavender Lassie	3	3.13
137	Lolita	1	2.38	110	Windrose	3	1.04
141	Sunset Boulevard	1	0.75	114	Venice	3	1.09
1	Parole	2	2.5	115	Focus	3	1.63
5	Superstar	2	1.15	116	Simply	3	1.11
38	Auslo	2	3.25	4	Nemo	4	0.21
39	Ausmas	2	1.69	32	Mevrouv Nath. Nypels	4	1.03
41	La Sevillana	2	1.16	131	Cute Haze	4	0.63
43	Rumba	2	1.07				

Suppl. Table S8. Validation of KASP marker Rh_FR_SNP201K with 282 independent rose cultivars**.**

Cultivar	Scent	SNP dosage	Cultivar	Scent	SNP dosage	Cultivar	Scent	SNP dosage
Annelies	1	0	Sahara	1	1	Palmengarten Frankfurt	1	2
Artemis	3	0	Sangerhausen Jubiläumsrose	1	1	Paprika	1	2
Astrid von Hardenberg	3	0	Solero	1	1	Poesie	1	2
Bernstein-Rose	2	0	Sommerabend	1	1	Postillion	2	2
CF Meyer	3	0	Souvenier de Rose Marie	1	1	Pretty Pink	1	2
Claire Jolly	1	0	Sunny Sky	2	1	Red Flame	3	2
Diamant	1	0	The Mayflower	3	1	Romantic Roadrunner	2	2
Dinky	1	0	Tradition 95	1	1	Rosarium Uetersen	1	2
Easy to cut	1	0	Twins	2	1	Rose von Oldenburg	1	2
Fils des Saison	1	0	Valencia	2	1	Rosenfee	1	2
First Lady	1	0	Vodacom	1	1	Rosy Purple	1	2
Flash	1	0	Voyage	2	1	Rotilia	1	2
Fortissima	1	0	Dame de Coeur	1	1	Shanty	1	2
Frisson Frais	1	0	Acapella	3	2	Shirley	2	2
Garden of Roses	3	0	Alden Biesen	1	2	Sibelius	1	2
Goldener Olymp	2	0	Aloha	1	2	Silver River	1	2
La Perla	1	0	Amber Sun	1	2	Sirius	1	2
Lupo	1	0	Amulett	1	2	Smile	1	2
Medeo	1	0	Apache	1	2	Souvenir de Baden Baden	2	2
Memoire	2	0	Aphrodite	3	2	Speelwark	2	2
Mossue de 4 saisons	3	0	Ascot	2	2	Summer Memories	1	2
Ms Doreen Pike	2	0	Aspirin	1	2	Sunny Rose	1	2
Paloma	2	0	Ausrimini	3	2	T06622	1	2
Pascaline	2	0	Bad Birnbach	1	2	The Pilgrims	2	2
Perennial Blue	3	0	Barock	2	2	Uetersener Klosterrose	3	2
Puccini	1	0	Bassino	1	2	Uetersen. Rosenprinzessin	1	2
Pur Roadrunner	3	0	Bel Esprit	1	2	Vanilla Cream	1	2
Purple Lodge	3	0	Belvedere	1	2	Verdi	2	2
Romanze	2	0	Biedermeier Garden	3	2	Vif Eclat	1	2
Rosa rugosa Foxi	3	0	Bouquet Parfait	1	2	Walferdange	1	2
R. rugosa Schneeeule	3	0	Brichet	2	2	Walzertraum	2	2
Rosanna	1	0	Brigitte de Villenfagne	2	2	Wavria	1	2
Rosenstadt Freising	2	0	Caesera	2	2	White Haze	1	2
Scepter´s Isle	3	0	Camelot	2	2	Zaide	1	2
Schoone Gezelle Blomme	1	0	Canary	2	2	Antike 89	2	3
Waterloo	2	0	Candela	1	2	Aperitif	2	3
White Chrystal	2	0	Cap Diamant	3	2	Bad Wörishofen 2005	1	3
White Roadrunner	2	0	Capricia	3	2	Bienenweide gelb	1	3
Yellow light	2	0	Centro-Rose	1	2	Big Purple	2	3
Alabaster	1	1	Claus Dalby	2	2	Caramella	1	3
Alpenglühen	1	1	Cubana	1	2	Cherry Lips	1	3
Altera	3	1	Diamant Rose	1	2	Deep Impression	1	3
Amadeus	1	1	Dublin Bay	1	2	Dornröschenschloss Sababurg	1	3
Aprikola	1	1	Duftrausch	3	2	Elmshorn	1	3
Augusta Luise	3	1	Duftzauber 84	2	2	Fantasia Mondiale	1	3

Austriana	1	1	Eifelzauber	1	2	Giardina	2	3
Avec Amour	1	1	Elvis	3	2	Golden Silk	2	3
Aya Renaissance	2	1	Emil Nolde Rose	2	2	Goldmarie	1	3
Bienenweide apricot	1	1	Eskelund	3	2	Gräfin Sonja	1	3
Blühwunder 08	1	1	Fairest Cape	1	2	Grande Amore	1	3
Bremer Stadtmusikant	2	1	Felicita	3	2	Helga	1	3
Candleligh	2	1	Felicitas	1	2	Home + Garden	1	3
Catherine Laborde	2	1	Francoise Drion	1	2	Jaqueline Humery	1	3
Cherry Girl	1	1	Friedenslicht	1	2	Jour de Fetes	3	3
Christel von der Post	1	1	Geisha	1	2	Jugenliebe	1	3
Cinderella	1	1	Gelber Engel	1	2	Lilly Marleen	1	3
Delicia	2	1	Gentile Harmonie	3	2	Lions Rose	2	3
Eliza	1	1	Golden Gate	2	2	Mariandel	1	3
Escimo	1	1	Golden Medaillon	2	2	Marie-Luise Marjan	2	3
Feeling	1	1	Golden Wings	1	2	Meimelliac	3	3
Gartenspaß	1	1	Goldelse	1	2	Mirato	1	3
Golden Monica	1	1	Hans Gönewein Rose	1	2	Mondial	1	3
Golden Tower	2	1	Hansaland	1	2	Moonlight	2	3
Goldschatz	1	1	Harlekin	1	2	Pearl Mirato	1	3
Grete Schichedanz	2	1	Harlow Carr	3	2	Princ. Josephine-Charlotte	1	3
Heavenly Pink	1	1	Helmut Kohl Rose	1	2	Purple Kiss	1	3
Helene de Gerlach	1	1	Inka	2	2	Racquel	3	3
Hella	1	1	Internescori	3	2	Rodeo	1	3
Kahlua	1	1	Jet Spray	1	2	Rosario	1	3
Kupferkönigin	2	1	Juwena	1	2	Roter Korsar	1	3
Ladybird	1	1	Karl Ploberger	1	2	Rugelda	1	3
Magma	1	1	Laguna	2	2	Sana Rose	1	3
Manita	1	1	Lampion	1	2	Schloß Ippenburg	3	3
Maria Mathilda	1	1	Liebeszauber	2	2	Schöne Berlinerin	2	3
Maria Teresa	1	1	Maaseik	1	2	Schwarze Madonna	1	3
Marie Antoinette	2	1	Madame Bovary	3	2	Sommersonne	1	3
Matador	1	1	Mainaufeuer	1	2	Sonnenröschen	1	3
Melina	2	1	Marc Chagall	3	2	Soul	3	3
Mon Petit Chou	2	1	Marvelle	1	2	Vogelpark Walsrode	1	3
Ninetta	1	1	Matchball	1	2	White Magic	1	3
Parfume de Grasse	2	1	Maxim	3	2	Zwergfee 09	1	3
Pashmina	1	1	Meidomonac	1	2	Anda	1	4
Petticoat	2	1	Mein schöner Garten	1	2	Apricot Clementine	1	4
Piano	1	1	Moneta	1	2	Bukavu Lenbrirus	1	4
Pink Roadrunner	3	1	Morning Sun	1	2	Domstadt Fulda	1	4
Planten un Blomen	1	1	Mozart	1	2	Fortuna	1	4
Purple Haze	1	1	Natalie Renaissance	3	2	History	1	4
Rebell	1	1	NDR1 Radio Niedersachsen	1	2	Pastella	1	4
Red Haze	1	1	Neige d'Ete	1	2	Pink Bassino	1	4
Rody	1	1	Neon	1	2	Playful Rokoko	1	4
Romina	3	1	Olympisches Feuer 92	1	2	Pomponella	1	4
Rose de la Petit Chabote	1	1	Omi Oswald	1	2	Rosenprofessor Sieber	1	4
Rose de la Pierette	2	1	Osiana	1	2	Satina	1	4
Rotkäppchen	1	1	Paganini	1	2	Tornado	1	4

Suppl. Table S9. Significant SNPs associated with petal number in roses.

SNP-marker	Chr	Position (Mbp)	p-value	Effects[1)] (Box Cox)			Effects (nontransformed)			MAF	F	errordf	markerR2	Genetic Var	Residual Var	-2LnLikelihood
				AA	AB	BB	AA	AB	BB							
Rh_PN_SNP1	0	0.5	9.48E-22	0	1.23	-	0	6.93	-	0.43421	189.89270	72	0.00205	1.28508	1.29E-05	263.39344
Rh_PN_SNP2	0	25.4	1.22E-06	0	-1.05	-	0	2.04	-	0.40196	30.95576	48	0.27027	1.24473	0.03613	165.78737
Rh_PN_SNP3	0	31.1	9.94E-18	0	1.45	-	0	13.36	-	0.30159	150.07603	59	0.00204	1.61734	1.62E-05	227.52784
Rh_PN_SNP4	1	13.9	3.45E-49	0.35	0	-	5.75	0	-	0.26812	1883.65696	65	0.38356	1.18941	1.19E-05	231.70061
Rh_PN_SNP5	1	26.6	1.69E-06	0.45	0	-	9.91	0	-	0.11765	27.52006	64	0.28266	1.62062	0.00737	243.24663
Rh_PN_SNP6	1	53.2	9.21E-07	-1.04	0	-	-22.2	0	-	0.25789	27.78847	91	0.20940	1.30022	0.07861	325.57573
Rh_PN_SNP7	2	0.7	6.08E-49	0.53	0	-	5.06	0	-	0.375	2296.39532	60	0.25416	1.38046	1.38E-05	223.28086
Rh_PN_SNP8	2	14.5	2.09E-07	-	0	1.05	-	0	6.35	0.68217	31.92032	86	0.24129	1.53180	0.04896	318.25510
Rh_PN_SNP9	2	15.4	2.03E-56	0	-0.35	-	0	-2.93	-	0.30769	2223.44512	74	0.49884	1.31386	1.31E-05	273.07098
Rh_PN_SNP10	2	32	4.76E-43	-0.42	-0.35	0	-15.62	-13.63	0	0.32110	1139.23189	52	0.62001	1.59100	1.59E-05	199.91830
Rh_PN_SNP11	2	63.5	3.24E-09	-	-1.4	0	-	-28.9	0	0.55118	46.87350	66	0.00121	1.44613	1.45E-05	249.13878
Rh_PN_SNP12	2	72.9	4.47E-55	0	-0.53	-	0	-5.25	-	0.16667	2037.05951	74	0.27212	1.67231	1.67E-05	289.92615
Rh_PN_SNP13	3	4.2	8.85E-07	-	-1.1	0	-	-8.22	0	0.51923	28.65996	77	0.21471	1.31621	0.04304	274.84683
Rh_PN_SNP14	3	4.3	7.49E-14	0	-1.22	-	0	-6.09	-	0.43182	124.47396	41	0.00166	1.59122	1.59E-05	155.42742
Rh_PN_SNP15	3	7.7	2.03E-11	0	-1.23	-	0	-7.45	-	0.37838	63.94858	70	0.28086	1.56024	0.00817	265.33796
Rh_PN_SNP16	3	12.4	2.80E-08	0	0.4	1.5	0	12.13	29.48	0.47445	22.80167	68	0.35618	1.53132	0.01422	261.16380
Rh_PN_SNP17	3	33.2	1.67E-06	4.31	2.35	0	58.16	20.32	0	0.48980	16.09709	73	0.22308	0.19757	1.36721	243.56026
Rh_PN_SNP18	3	38.6	1.43E-06	0	2.43	4.26	0	23.16	58.48	0.45283	16.10501	78	0.24249	0.31944	1.40862	272.47126
Rh_PN_SNP19	3	39.5	1.27E-10	0	-1.16	-	0	-12.08	-	0.42553	70.65561	44	0.56265	1.19252	0.01105	153.12126
Rh_PN_SNP20	3	41.1	2.17E-08	1.06	0	-	7.08	0	-	0.37791	38.32138	82	0.27135	1.50732	0.03771	304.72587
Rh_PN_SNP21	3	42	4.32E-08	-	-1.06	0	-	-10.41	0	0.58553	36.21930	86	0.25500	1.31799	0.04159	308.94159
Rh_PN_SNP22	3	42.3	2.67E-57	-0.36	0	-	-5.47	0	-	0.27703	2636.59510	71	0.63493	1.41515	1.42E-05	265.29582
Rh_PN_SNP23	3	42.5	6.87E-97	0	0.1	1.23	0	-4.89	13.46	0.49342	12856.12493	77	0.54354	1.67237	1.67E-05	296.22960
Rh_PN_SNP24	4	30	1.72E-08	1.13	0	-	14.02	0	-	0.21324	41.56574	65	0.31630	1.53222	0.03137	241.13634
Rh_PN_SNP25	4	30.4	2.40E-45	-	0	0.35	-	0	4.57	0.65116	2301.54481	54	0.59213	1.50369	1.50E-05	204.30888
Rh_PN_SNP26	4	53.9	1.54E-06	-	-0.44	0	-	-20.11	0	0.55224	27.66080	70	0.26437	1.46749	0.00732	260.56948
Rh_PN_SNP27	4	58	4.05E-60	-	0.53	0	-	7.5	0	0.57857	2447.89721	78	0.28585	1.46017	1.46E-05	295.51415
Rh_PN_SNP28	5	9	7.82E-07	-	-1.41	0	-	-35.42	0	0.52727	31.34386	54	0.29835	1.49740	0.01178	206.22392
Rh_PN_SNP29	5	11	3.20E-80	-	-1.13	0	-	-14.74	0	0.63725	24773.42944	61	0.54149	1.72879	1.73E-05	234.10257
Rh_PN_SNP30	5	18.7	1.18E-08	-	1.08	0	-	11.6	0	0.83486	39.69028	88	0.26048	1.46150	0.03326	328.23132
Rh_PN_SNP31	5	31	9.31E-07	-	0	1.01	-	0	7.54	0.55862	28.45850	78	0.22743	1.34831	0.04858	278.03087
Rh_PN_SNP32	5	55.9	4.71E-49	-	-0.35	0	-	0.75	0	0.67368	2316.95159	60	0.62012	1.56720	1.57E-05	230.42282
Rh_PN_SNP33	5	59.1	1.57E-06	-	0.43	0	-	7.11	0	0.77528	27.92922	66	0.28246	1.26406	0.00701	234.87151
Rh_PN_SNP34	5	63.6	5.50E-13	-0.54	-1.16	0	-8.83	-20.19	0	0.47328	45.68145	64	0.53595	1.38478	0.00822	234.87561
Rh_PN_SNP35	5	67.3	6.37E-50	-	0	0.35	-	0	9.5	0.74118	2367.83933	61	0.61766	1.52751	1.53E-05	231.06069

Rh_PN_SNP36	5	77	1.10E-09	0	-1.12	-	0	-9.02	-	0.34783	50.66532	65	0.36522	1.63115	0.03000	241.15639
Rh_PN_SNP37	6	1.5	3.20E-53	-0.35	0	-	-4.81	0	-	0.22917	2240.03698	68	0.60516	1.58643	1.59E-05	261.93451
Rh_PN_SNP38	6	13.6	6.27E-52	0	0.35	-	0	8.22	-	0.21429	2213.26467	66	0.61478	1.63123	1.63E-05	256.31370
Rh_PN_SNP39	6	19.2	2.48E-08	0	-1.15	-	0	1.39	-	0.44521	39.74650	69	0.32034	1.68738	0.03860	256.57150
Rh_PN_SNP40	6	52	7.32E-07	1.22	0	-	33.64	0	-	0.26119	30.26814	64	0.30149	1.26677	0.04246	227.56310
Rh_PN_SNP41	6	57	2.15E-06	-	1.02	0	-	16	0	0.73846	25.70391	92	0.19534	1.33354	0.07658	329.46576
Rh_PN_SNP42	6	58.2	4.44E-60	-	-0.35	0	-	-9.51	0	0.57554	2618.45852	76	0.63706	1.42926	1.43E-05	285.91024
Rh_PN_SNP43	6	67.2	2.00E-42	0	0.35	-0.49	0	-1.82	-9.65	0.56522	1018.13864	53	0.47925	1.37451	1.37E-05	195.58659
Rh_PN_SNP44	7	7.8	7.71E-09	-	1.02	0	-	16	0	0.13483	41.22348	85	0.29084	1.57254	0.03743	318.15097
Rh_PN_SNP45	7	20.7	1.45E-62	-	0	-0.36	-	0	-0.07	0.81905	2337.15347	84	0.60324	1.52205	1.52E-05	322.22456
Rh_PN_SNP46	7	40.9	3.40E-63	0	-0.61	-	0	-7.61	-	0.28571	13129.54454	52	0.79816	1.16879	1.17E-05	184.20884
Rh_PN_SNP47	7	53.8	6.71E-47	0	0.35	-	0	13.39	-	0.27778	3171.37443	51	0.60087	1.10259	1.10E-05	175.54324

[1)] The effect of the genotypic class with the lowest frequency is set to zero. The effects of the other genotypic classes are given as deviations between their estimated values and the lowest frequency class.

Suppl. Table S10. Significant SNPs associated with petal length in roses.

SNP-marker	Chr	Position (Mbp)	p-value	Effects			MAF	F	errordf	markerR2	Genetic Var	Residual Var	-2LnLikelihood
				A:A	A:B	B:B							
Rh_PL_SNP1	0	0.1	7.22E-07	13.22	8.85	0	0.35088	16.77835	86	0.23161	31.99041	8.61594	579.81215
Rh_PL_SNP3	1	64.3	1.78E-07	0	8.89	-	0.36709	33.22099	75	0.24785	30.79522	9.03166	506.42776
Rh_PL_SNP2	1	64.3	2.29E-07	-	8.89	0	0.56688	31.74548	85	0.21113	27.11305	10.60303	569.75607
Rh_PL_SNP4	1	64.3	2.86E-07	-	8.56	0	0.57778	31.97832	74	0.22821	29.13684	8.85313	495.45991
Rh_PL_SNP6	1	64.5	2.39E-07	-	8.72	0	0.57778	32.39115	75	0.21518	25.23308	9.96849	496.92794
Rh_PL_SNP7	1	64.5	2.97E-07	-	8.32	0	0.57778	31.78117	75	0.19920	22.21971	9.55375	487.08433
Rh_PL_SNP5	1	64.5	9.98E-07	0	8.16	-	0.38333	27.82372	86	0.18961	27.79586	11.51079	577.14941
Rh_PL_SNP8	1	64.6	3.99E-07	0	8.99	-	0.38235	30.52031	81	0.21577	30.36635	10.46215	547.84588
Rh_PL_SNP9	1	64.7	8.04E-07	-	8.21	0	0.5625	28.38064	86	0.18782	26.07880	11.23725	574.01234
Rh_PL_SNP10	1	64.7	9.97E-07	0	8.11	-	0.38095	28.15665	80	0.17181	21.39664	11.93274	524.59463
Rh_PL_SNP11	1	64.7	1.00E-06	0	7.96	-	0.38636	27.92177	84	0.17278	22.51358	11.15889	553.67579
Rh_PL_SNP12	2	65.6	1.34E-06	21.39	9.64	0	0.50667	16.20516	78	0.19527	15.17913	20.39553	510.68557
Rh_PL_SNP13	2	67	1.58E-06	0	7.49	17.53	0.45775	16.17306	73	0.25384	28.96014	9.11260	487.72703
Rh_PL_SNP14	2	68.7	7.45E-08	21.84	12.02	0	0.25850	20.94074	71	0.29102	26.55237	7.87423	470.56206
Rh_PL_SNP15	3	34.8	6.36E-07	0	8.37	12.05	0.44079	17.36211	76	0.25951	33.40497	8.11468	513.87081
Rh_PL_SNP16	3	36	4.86E-07	-8.68	1.1	0	0.47682	17.75595	76	0.27138	37.83200	7.09572	517.11428
Rh_PL_SNP17	3	36.9	1.60E-06	-	7.78	0	0.57037	27.34375	73	0.22283	34.33311	9.33555	495.52926
Rh_PL_SNP18	3	42.7	1.48E-06	0	-4.15	-13.97	0.62931	27.77305	70	0.23412	31.75840	10.84623	472.38130
Rh_PL_SNP19	3	64.6	6.99E-07	0	0.86	-9.8	0.46626	17.00976	81	0.25006	35.62380	7.55399	553.04266
Rh_PL_SNP20	4	56.1	1.14E-07	2.82	0	-	0.26415	38.45997	50	0.11056	35.72104	0.11662	334.43349
Rh_PL_SNP22	5	10.5	8.15E-07	-	10.68	0	0.59375	28.10049	91	0.18650	28.77672	10.60413	611.20469
Rh_PL_SNP21	5	10.5	1.20E-06	-	10.9	0	0.61029	27.73223	79	0.20616	30.71745	10.60756	530.35257
Rh_PL_SNP23	5	10.9	2.32E-07	-	12.17	0	0.60584	32.13911	79	0.23306	30.88788	10.02637	529.47389
Rh_PL_SNP24	5	10.9	1.39E-06	-	11.71	0	0.29221	27.65399	74	0.21839	30.98776	10.65629	496.39716
Rh_PL_SNP25	5	11.1	2.77E-07	0	11.62	-	0.33523	31.29625	84	0.21595	28.68533	10.51656	563.67109
Rh_PL_SNP27	5	11.4	8.50E-08	0	12.24	-	0.32738	34.84286	80	0.24834	29.10187	9.96903	534.06046
Rh_PL_SNP26	5	11.4	4.69E-07	0	11.76	-	0.29054	30.94442	70	0.24419	31.50258	10.26391	468.14646
Rh_PL_SNP28	5	12.7	6.86E-07	0	12.82	-	0.33133	29.20796	79	0.21686	30.21024	10.36051	530.72110
Rh_PL_SNP29	5	12.7	7.62E-07	0	10.77	-	0.31176	28.80297	81	0.20767	30.06434	10.69255	543.50118
Rh_PL_SNP30	5	12.8	9.92E-08	0	11.09	-	0.33333	33.72199	89	0.21888	27.34319	9.36825	592.65074
Rh_PL_SNP31	5	12.8	1.65E-07	0	11.11	-	0.32447	32.29508	90	0.20995	27.86633	10.28847	602.35214
Rh_PL_SNP32	5	12.8	3.23E-07	-	11.06	0	0.61379	30.82452	85	0.21081	29.65405	10.44296	570.38159
Rh_PL_SNP34	5	13	2.69E-08	-	12.35	0	0.625	39.20242	71	0.28975	28.22676	8.97360	467.57646
Rh_PL_SNP33	5	13	1.48E-06	-	12.97	0	0.47059	16.10072	77	0.24434	35.89935	7.85895	525.12707
Rh_PL_SNP36	5	13.1	3.37E-07	-	10.65	0	0.625	30.91074	82	0.20946	26.80069	10.58597	544.73056
Rh_PL_SNP37	5	13.1	3.63E-07	0	10.63	-	0.61806	30.51455	85	0.20408	27.54098	10.66115	566.42027

Rh_PL_SNP35	5	13.1	1.20E-06	0	10.01	-	0.30899	27.39440	85	0.18983	26.78510	10.87583	565.30094
Rh_PL_SNP39	5	13.3	1.05E-07	0	11.47	-	0.30357	34.24903	80	0.23055	27.04455	10.17389	530.35985
Rh_PL_SNP38	5	13.3	2.20E-07	0	11.31	-	0.30588	32.05896	82	0.21758	27.18023	11.18767	548.00266
Rh_PL_SNP40	5	13.7	5.44E-07	-	12.74	0	0.61789	30.27395	73	0.23075	29.09507	11.06721	488.21371
Rh_PL_SNP41	5	13.8	2.35E-07	0	12.18	-	0.31579	32.70865	72	0.24029	26.73281	10.64248	479.49963
Rh_PL_SNP42	5	13.8	1.52E-06	0	11.11	-	0.28947	27.49138	73	0.21193	30.98569	10.58253	488.20452
Rh_PL_SNP45	5	14.1	1.27E-06	0	10.47	-	0.32222	27.20660	86	0.18975	29.52243	10.86645	579.20059
Rh_PL_SNP44	5	14.1	1.34E-06	0	10.56	-	0.60811	27.07242	86	0.18743	29.36577	10.85967	578.77927
Rh_PL_SNP43	5	14.1	1.39E-06	0	10.97	-	0.608	27.78742	72	0.23126	33.42963	11.06265	491.51836
Rh_PL_SNP46	5	14.4	1.27E-06	0	12.06	-	0.32738	27.51988	80	0.19660	28.69775	10.55716	536.19412
Rh_PL_SNP47	5	14.5	1.01E-06	0	10.82	-	0.31176	28.06978	81	0.19963	29.08049	10.77730	541.77992
Rh_PL_SNP49	5	14.5	1.16E-06	0	10.86	-	0.31034	27.58177	83	0.19903	31.34856	10.71608	560.09770
Rh_PL_SNP48	5	14.5	1.56E-06	-	10.2	0	0.61111	26.77613	84	0.18390	27.21955	10.88118	560.29460
Rh_PL_SNP50	5	14.8	1.55E-06	-	10.24	0	0.61940	27.00106	80	0.19467	28.44473	10.55846	535.72295
Rh_PL_SNP52	5	14.9	2.36E-07	0	8.81	-	0.33133	32.09512	79	0.23260	32.11153	8.98740	531.18734
Rh_PL_SNP54	5	14.9	5.51E-07	0	8.55	-	0.58861	29.19867	89	0.19522	28.96967	10.50274	597.47807
Rh_PL_SNP53	5	14.9	7.82E-07	-	8.46	0	0.58861	28.29850	89	0.19210	29.83268	10.51207	599.38590
Rh_PL_SNP51	5	14.9	1.22E-06	0	8.11	-	0.33721	27.51774	82	0.18178	26.06756	10.26116	543.81779
Rh_PL_SNP56	5	15	6.62E-08	0	9.44	-	0.30263	36.38068	72	0.25051	27.63242	8.24974	474.35650
Rh_PL_SNP55	5	15	2.47E-07	0	8.94	-	0.33721	31.75087	82	0.22163	28.95361	10.28337	548.69179
Rh_PL_SNP59	5	15.1	4.93E-09	0	9.86	-	0.33333	42.42048	86	0.27737	31.22759	7.21802	577.92206
Rh_PL_SNP57	5	15.1	7.59E-08	0	11.09	-	0.31098	35.34968	78	0.24093	30.77830	8.75202	523.96755
Rh_PL_SNP58	5	15.1	4.08E-07	0	8.55	-	0.33152	30.03364	88	0.20247	28.92946	10.49713	591.36345
Rh_PL_SNP60	5	15.4	8.66E-07	0	8.31	-	0.32468	29.00069	73	0.22418	28.04814	11.88595	487.34667
Rh_PL_SNP61	5	16.1	1.57E-09	14.63	10.86	0	0.50355	26.78751	77	0.33727	27.21165	7.30505	506.77755
Rh_PL_SNP62	5	20.5	8.53E-07	11.59	9.63	0	0.5	17.28328	69	0.27981	33.98908	6.36232	465.82766
Rh_PL_SNP63	5	61.2	1.35E-06	0	8.01	-	0.33152	26.95820	88	0.18724	30.22658	10.65014	595.73359
Rh_PL_SNP64	5	61.8	1.74E-07	0	5.6	-4.65	0.47287	20.04088	65	0.33040	44.10809	3.63651	453.19245
Rh_PL_SNP65	6	16.5	9.09E-08	0	10.36	-	0.45070	36.05650	67	0.27795	33.94573	6.98072	450.78719
Rh_PL_SNP66	6	59.1	7.10E-12	0	4.44	-	0.44915	75.91221	55	0.19306	37.13230	0.11908	371.44245
Rh_PL_SNP67	6	62.2	1.64E-06	0	9.08	-	0.31111	26.54983	86	0.18084	28.52696	9.43016	575.46635
Rh_PL_SNP68	7	40.9	1.25E-20	0	0.46	-	0.28571	232.33177	52	0.72046	40.43295	4.04E-04	363.38526

[1)] The effect of the genotypic class with the lowest frequency is set to zero. The effects of the other genotypic classes are given as deviations between their estimated values and the lowest frequency class.

Suppl. Table S11. Significant SNPs associated with fragrance in roses.

SNP-marker	Chr	Position (Mbp)	p-value	Effects[1)] (Box Cox)			Effects (nontransformed)			MAF	F	errordf	markerR2	Genetic Var	Residual Var	-2LnLikelihood
				AA	AB	BB	AA	AB	BB							
Rh_FR_SNP1	0	10.7	6.52E-07	1.08	0.24	0	0.95	-0.03	0	0.23494	17.10799	81	0.23736	0.16581	0.06868	137.76845
Rh_FR_SNP2	0	19.6	4.15E-07	-	0	0.77	-	0	0.94	0.19565	29.99250	88	0.19681	0.07982	0.17732	146.41922
Rh_FR_SNP3	0	22.5	1.36E-08	-	0	0.82	-	0	1.01	0.65909	39.68718	84	0.24719	0.08527	0.15240	134.80363
Rh_FR_SNP7	0	22.5	2.11E-08	0.75	0	-	0.93	0	-	0.25806	37.96701	89	0.23242	0.10643	0.11526	140.62790
Rh_FR_SNP6	0	22.5	2.70E-07	0.66	0	-	0.85	0	-	0.26344	31.06211	89	0.19553	0.10253	0.10969	137.85349
Rh_FR_SNP5	0	22.5	7.32E-07	-	0	0.73	-	0	0.89	0.71	29.78908	69	0.21923	0.10159	0.08521	99.84660
Rh_FR_SNP4	0	22.5	1.51E-06	-	0	0.69	-	0	0.88	0.64706	26.81799	85	0.18194	0.10250	0.12403	135.53351
Rh_FR_SNP9	0	22.7	1.96E-09	-	0	0.83	-	0	1.04	0.68595	45.97665	80	0.26505	0.03416	0.20489	119.94471
Rh_FR_SNP8	0	22.7	6.44E-08	-	0	0.78	-	0	0.96	0.65185	35.19516	85	0.23018	0.08348	0.15997	137.46156
Rh_FR_SNP10	0	22.8	4.46E-10	-	0	0.97	-	0	1.11	0.63846	50.65273	80	0.31827	0.13016	0.06483	122.49333
Rh_FR_SNP11	0	22.8	9.23E-07	-	0	0.74	-	0	0.86	0.64394	28.24059	82	0.20274	0.12994	0.06229	124.87381
Rh_FR_SNP18	0	22.9	4.41E-09	0.79	0	-	0.98	0	-	0.25269	42.42298	89	0.24296	0.06996	0.16748	138.95309
Rh_FR_SNP19	0	22.9	7.46E-09	-	0	0.78	-	0	0.95	0.67742	41.77661	81	0.24888	0.09407	0.09627	115.88475
Rh_FR_SNP16	0	22.9	2.08E-08	0.76	0	-	0.93	0	-	0.25269	38.01246	89	0.22642	0.07134	0.17817	143.38233
Rh_FR_SNP14	0	22.9	6.11E-08	0.73	0	-	0.85	0	-	0.25556	35.26130	86	0.19904	0.00799	0.28398	139.28577
Rh_FR_SNP17	0	22.9	1.82E-07	0.72	0	-	0.94	0	-	0.25843	32.36528	85	0.20493	0.09735	0.11587	131.85781
Rh_FR_SNP15	0	22.9	3.17E-07	-	0	0.74	-	0	0.93	0.20556	30.81096	86	0.20982	0.11374	0.12409	142.54458
Rh_FR_SNP13	0	22.9	3.95E-07	0.71	0	-	0.82	0	-	0.26667	30.23218	86	0.18200	0.02559	0.27235	144.45577
Rh_FR_SNP12	0	22.9	1.01E-06	0.65	0	-	0.81	0	-	0.22840	28.25208	78	0.19190	0.09271	0.12041	119.30429
Rh_FR_SNP20	0	73	1.15E-06	-	0	0.77	-	0	1	0.65323	27.89374	78	0.19971	0.05757	0.22757	133.55057
Rh_FR_SNP21	1	5.1	6.60E-31	0	-1.49	-1.59	0	-0.85	-0.99	0.56923	208.49572	74	0.54585	0.24860	0.00223	131.07617
Rh_FR_SNP22	1	12.6	2.48E-07	-	0.85	0	-	1.08	0	0.83333	31.11039	92	0.19755	0.15323	0.07498	153.22870
Rh_FR_SNP23	1	24.9	1.00E-12	-	0.24	0	-	0.01	0	0.62025	97.40812	45	0.02132	0.21335	2.13E-06	81.00801
Rh_FR_SNP24	1	61.8	8.39E-58	-0.12	0	-	-0.08	0	-	0.22143	3388.563	66	0.75373	0.19957	2.00E-06	108.66187
Rh_FR_SNP25	1	64.6	1.10E-15	-	0	0.25	-	0	0.34	0.84	116.50097	61	0.52976	0.22084	5.07E-05	103.96366
Rh_FR_SNP26	2	2.2	2.58E-18	1.41	0.13	0	1.25	0.14	0	0.58824	70.62034	80	0.46820	0.22844	0.00909	135.25460
Rh_FR_SNP27	2	6.3	2.38E-07	0	-0.84	-0.8	0	-0.65	-0.8	0.50714	18.93378	74	0.24023	0.26386	0.01004	147.21143
Rh_FR_SNP28	2	62.3	6.29E-08	0	0.74	-	0	0.86	-	0.22396	34.74569	92	0.21271	0.13829	0.08295	149.52234
Rh_FR_SNP29	2	62.4	7.75E-08	0	0.69	-	0	0.86	-	0.22581	34.38920	89	0.21551	0.13013	0.09107	142.54325
Rh_FR_SNP30	2	62.7	1.26E-07	0	0.27	1.1	0	0.06	0.96	0.60993	19.13442	89	0.23649	0.15915	0.05740	143.41267
Rh_FR_SNP36	2	63.4	2.48E-07	-	0.75	0	-	0.87	0	0.65672	31.52521	85	0.20701	0.12979	0.06781	128.24134
Rh_FR_SNP31	2	63.4	4.24E-07	0	0.71	-	0	0.81	-	0.27368	29.77132	91	0.18952	0.13088	0.06521	137.89539
Rh_FR_SNP33	2	63.4	4.24E-07	0	0.71	-	0	0.81	-	0.27368	29.77132	91	0.18952	0.13088	0.06521	137.89539
Rh_FR_SNP34	2	63.4	4.24E-07	0	0.71	-	-	0	0.81	0.27368	29.77132	91	0.18952	0.13088	0.06521	137.89539
Rh_FR_SNP35	2	63.4	4.24E-07	0	0.71	-	0	0.81	-	0.27368	29.77132	91	0.18952	0.13088	0.06521	137.89539

Rh_FR_SNP32	2	63.4	6.43E-07	0	0.74	-	0	0.86	-	0.26374	28.90437	87	0.19345	0.13506	0.06804	134.16221
Rh_FR_SNP37	2	63.4	8.67E-07	-	0.7	0	-	0.8	0	0.64583	27.98841	90	0.18256	0.13293	0.06501	138.13021
Rh_FR_SNP39	2	63.5	8.33E-08	0	0.86	-	0	0.93	-	0.21429	36.45552	66	0.28182	0.13976	0.08801	105.35738
Rh_FR_SNP38	2	63.5	1.49E-06	-	0	0.86	-	0	0.98	0.74286	27.39679	75	0.20492	0.14260	0.07859	121.78587
Rh_FR_SNP40	2	63.7	1.35E-06	0.76	0	-	0.78	0	-	0.18889	27.05700	86	0.18280	0.14549	0.07572	139.22934
Rh_FR_SNP41	2	64.2	1.56E-06	1.09	0.27	0	1.31	0.42	0	0.24294	15.68160	87	0.20661	0.15360	0.08033	145.63847
Rh_FR_SNP42	2	65	9.79E-07	-0.71	0	-	-0.72	0	-	0.27128	27.67871	90	0.18388	0.15143	0.08654	152.68690
Rh_FR_SNP43	2	65.2	7.13E-07	-0.76	0	-	-0.69	0	-	0.29670	28.63908	87	0.19542	0.16025	0.08025	148.93746
Rh_FR_SNP44	2	65.6	3.63E-07	-	0	-0.75	-	0	-0.75	0.64789	30.22727	90	0.19591	0.15070	0.08217	150.91083
Rh_FR_SNP45	2	65.8	6.00E-07	-0.77	0	-	-0.79	0	-	0.24118	29.43431	81	0.20734	0.15485	0.08188	134.52834
Rh_FR_SNP47	2	65.9	7.91E-10	1.3	0.33	0	1.41	0.32	0	0.22293	27.96448	77	0.32698	0.14084	0.04414	111.09097
Rh_FR_SNP46	2	65.9	6.65E-08	1.19	0.38	0	1.23	0.36	0	0.23313	20.52039	80	0.26135	0.15483	0.04828	124.64723
Rh_FR_SNP48	2	66	4.02E-07	-	0	0.72	-	0	0.81	0.72358	30.18562	86	0.19858	0.10908	0.12921	143.71486
Rh_FR_SNP50	2	66.5	1.31E-09	-	0	0.93	-	0	1.01	0.67692	46.51190	85	0.28506	0.14172	0.06451	133.59395
Rh_FR_SNP49	2	66.5	1.44E-06	-	0.66	0	-	0.73	0	0.63333	26.62015	92	0.17303	0.13762	0.10019	155.13073
Rh_FR_SNP51	2	66.7	4.63E-07	0.9	0	-	0.98	0	-	0.2	30.89037	71	0.24055	0.15503	0.04485	110.09588
Rh_FR_SNP52	2	66.7	9.87E-07	-	0	0.72	-	0	0.83	0.69466	27.75218	88	0.19016	0.14359	0.09327	149.22680
Rh_FR_SNP53	2	66.7	1.20E-06	-	0	0.69	0	0.82	-	0.72358	27.35559	86	0.19196	0.13430	0.10187	144.38870
Rh_FR_SNP54	2	66.9	8.46E-08	-	0	0.83	-	0	0.87	0.67442	34.52075	84	0.23541	0.14273	0.07239	136.09644
Rh_FR_SNP55	2	67	2.58E-07	-	0	0.75	-	0	0.84	0.71774	31.36017	86	0.21165	0.13241	0.10055	144.12853
Rh_FR_SNP56	2	67.6	2.42E-09	0.9	0	-	1.03	0	-	0.22941	45.17798	81	0.29795	0.13547	0.07028	125.99980
Rh_FR_SNP57	2	67.9	4.15E-07	0.71	0	-	0.85	0	-	0.67769	30.55414	79	0.21043	0.05121	0.24904	136.40979
Rh_FR_SNP58	2	67.9	9.31E-07	0.74	0	-	0.91	0	-	0.20253	28.65896	75	0.21194	0.07230	0.19949	125.85118
Rh_FR_SNP62	2	68.1	8.86E-08	-	0	0.84	-	0	0.95	0.66929	34.55508	82	0.23332	0.12633	0.05649	119.89669
Rh_FR_SNP61	2	68.1	6.61E-07	-	0	0.84	-	0	1.02	0.68696	29.51328	76	0.22055	0.08006	0.18605	128.72135
Rh_FR_SNP60	2	68.1	1.69E-06	0.7	0	-	0.83	0	-	0.18539	26.52453	85	0.18529	0.08832	0.18186	144.99687
Rh_FR_SNP59	2	68.1	1.71E-06	0.75	0	-	0.85	0	-	0.15854	26.85739	78	0.18528	0.07650	0.19104	131.13660
Rh_FR_SNP63	2	68.8	3.17E-07	0.71	0	-	0.86	0	-	0.70370	31.68317	74	0.21968	0.05813	0.20109	119.59107
Rh_FR_SNP64	2	69	6.21E-07	-	0	0.71	-	0	0.88	0.69841	29.10643	85	0.20565	0.10547	0.14141	142.29891
Rh_FR_SNP65	2	69.8	3.12E-09	0.93	0	-	1.14	0	-	0.17123	46.17916	70	0.28667	0.03904	0.19408	103.47006
Rh_FR_SNP66	2	69.8	5.94E-09	0.8	0	-	1.03	0	-	0.25269	41.56258	89	0.23318	0.04455	0.21935	142.28022
Rh_FR_SNP68	2	69.9	9.82E-12	-	0	1.08	-	0	1.32	0.65041	64.41586	77	0.37507	0.12974	0.04874	110.55066
Rh_FR_SNP67	2	69.9	6.68E-10	0.82	0	-	1.01	0	-	0.23889	48.42807	86	0.25243	0.01628	0.25330	134.48049
Rh_FR_SNP70	2	70.1	3.10E-11	0.91	0	-	1.15	0	-	0.22727	58.65346	84	0.30951	0.03799	0.19408	124.37349
Rh_FR_SNP80	2	70.1	3.84E-11	-	0	0.85	-	0	1.07	0.66912	57.15464	88	0.29077	0.02420	0.22066	131.54844
Rh_FR_SNP79	2	70.1	1.96E-10	-	0	0.87	-	0	1.09	0.67669	52.10801	87	0.27782	0.04843	0.19231	132.94037
Rh_FR_SNP77	2	70.1	2.06E-10	0.83	0	-	1.06	0	-	0.24737	51.35215	91	0.26693	0.05198	0.18032	136.91347
Rh_FR_SNP69	2	70.1	3.84E-10	-	0	0.86	-	0	1.09	0.67717	50.61590	83	0.28033	0.02968	0.22922	128.74135
Rh_FR_SNP76	2	70.1	3.86E-10	0.84	0	-	1.06	0	-	0.23626	49.97793	87	0.26673	0.04647	0.18893	130.83245

Rh_FR_SNP72	2	70.1	4.01E-10	0.85	0	-	1.1	0	-	0.21429	50.99438	80	0.29578	0.03463	0.19006	115.30116
Rh_FR_SNP75	2	70.1	4.97E-10	0.83	0	-	1.06	0	-	0.23913	49.05593	88	0.26566	0.05489	0.18136	135.07131
Rh_FR_SNP78	2	70.1	5.56E-10	0.81	0	-	1.04	0	-	0.24176	48.85332	87	0.26791	0.03746	0.21441	133.95955
Rh_FR_SNP74	2	70.1	5.92E-10	-	0	0.86	-	0	1.04	0.67164	48.66155	87	0.24714	2.92E-06	0.29195	138.17936
Rh_FR_SNP73	2	70.1	9.27E-10	0.84	0	-	1.02	0	-	0.24457	47.15767	88	0.23975	2.92E-06	0.29193	140.78373
Rh_FR_SNP71	2	70.1	1.42E-09	0.81	0	-	1.03	0	-	0.23626	46.00979	87	0.25372	0.05544	0.17718	131.38598
Rh_FR_SNP82	2	70.1	4.17E-09	-	0.36	0	1.62	0.52	0	0.33588	26.48211	65	0.35502	0.12194	0.07749	96.32473
Rh_FR_SNP81	2	70.1	3.92E-07	0.8	0	1.3	1.06	0	-	0.35632	30.43895	83	0.20890	0.08714	0.16170	136.20452
Rh_FR_SNP84	2	70.2	2.03E-10	-	0	0.82	-	0	1.04	0.66912	51.83872	88	0.26408	0.02696	0.22147	133.33732
Rh_FR_SNP83	2	70.2	4.23E-10	-	0	0.83	-	0	1.08	0.68217	49.84079	86	0.27375	0.04833	0.18426	129.43681
Rh_FR_SNP88	2	70.2	4.22E-09	0.83	0	-	1.02	0	-	0.23563	43.10924	84	0.23979	0.02659	0.24650	134.42567
Rh_FR_SNP86	2	70.2	8.94E-09	0.79	0	-	1.01	0	-	0.25532	40.29577	90	0.22601	0.04548	0.21737	144.78739
Rh_FR_SNP85	2	70.2	4.31E-08	0.76	0	-	0.99	0	-	0.26344	35.98884	89	0.20986	0.04118	0.23950	146.54519
Rh_FR_SNP87	2	70.2	6.21E-08	0.82	0	-	-	1.05	0	0.29114	36.22972	75	0.24942	3.13E-06	0.31312	124.07686
Rh_FR_SNP90	2	70.4	2.47E-11	-	0	0.88	-	0	1.12	0.67407	58.60634	88	0.30049	0.04370	0.18006	128.61998
Rh_FR_SNP92	2	70.4	2.70E-10	-	0	0.83	-	0	1.07	0.66923	51.57242	84	0.29339	0.08863	0.09141	118.07222
Rh_FR_SNP89	2	70.4	3.10E-10	0.84	0	-	1.07	0	-	0.24194	50.36979	89	0.26852	0.05408	0.18125	135.20061
Rh_FR_SNP93	2	70.4	1.76E-09	-	0	0.81	-	0	1.03	0.67424	45.48488	86	0.25774	0.04889	0.19077	132.18964
Rh_FR_SNP91	2	70.4	2.28E-09	1	0	-	1.35	0	-	0.26829	45.79813	78	0.27560	0.05796	0.15402	113.12752
Rh_FR_SNP97	2	70.4	3.18E-09	-	0	0.8	-	0	0.99	0.65942	43.48545	88	0.23710	0.02719	0.24597	140.93885
Rh_FR_SNP96	2	70.4	6.50E-09	-	0	0.81	-	0	1.02	0.66917	41.61416	86	0.23962	0.04041	0.21366	134.63211
Rh_FR_SNP94	2	70.4	2.95E-07	-	0	0.73	-	0	0.95	0.62937	30.94270	87	0.19913	0.05313	0.22672	145.39276
Rh_FR_SNP95	2	70.4	1.34E-06	-	0	0.71	-	0	0.91	0.625	27.26801	82	0.18454	0.05477	0.23718	141.26284
Rh_FR_SNP98	2	70.5	2.78E-12	-	0	0.92	-	0	1.15	0.67669	66.22324	87	0.32096	0.03463	0.18380	122.43293
Rh_FR_SNP100	2	70.5	2.94E-09	0.85	0	-	1.08	0	-	0.21341	45.00912	78	0.26478	0.04665	0.19760	120.33364
Rh_FR_SNP99	2	70.5	1.57E-08	0.89	0	-	1.08	0	-	0.19178	41.16992	69	0.27047	0.08236	0.11183	98.28871
Rh_FR_SNP101	2	70.8	3.29E-07	-	0	0.72	-	0	0.88	0.65468	30.59903	88	0.19241	0.06809	0.19834	146.60847
Rh_FR_SNP102	2	70.9	9.39E-10	1	0	-	1.14	0	-	0.26875	48.96339	76	0.31878	0.14107	0.06408	118.42579
Rh_FR_SNP103	2	71.1	2.10E-11	0.99	0	-	1.1	0	-	0.28333	59.54917	86	0.32939	0.12580	0.05914	127.01111
Rh_FR_SNP104	2	71.1	2.93E-10	0.94	0	-	1.07	0	-	0.27841	51.31071	84	0.30800	0.13065	0.06066	125.35758
Rh_FR_SNP106	2	71.1	2.46E-09	0.9	0	-	0.99	0	-	0.27586	44.85245	83	0.28508	0.13797	0.06385	127.05854
Rh_FR_SNP105	2	71.1	2.77E-09	0.89	0	-	0.99	0	-	0.27059	44.76132	81	0.28992	0.14116	0.05489	123.02807
Rh_FR_SNP107	2	71.1	2.53E-08	0.74	0	-	0.9	0	-	0.25	37.54439	88	0.22444	0.07109	0.16229	137.20205
Rh_FR_SNP114	2	71.1	3.05E-08	-	0	0.74	-	0	0.92	0.65957	36.86371	90	0.21945	0.07846	0.16597	145.29744
Rh_FR_SNP113	2	71.1	3.08E-08	-	0	0.74	-	0	0.91	0.65734	36.75889	91	0.21705	0.07759	0.16549	145.40732
Rh_FR_SNP117	2	71.1	3.65E-08	0.74	0	-	0.92	0	-	0.25269	36.44317	89	0.22009	0.07985	0.16686	143.52119
Rh_FR_SNP118	2	71.1	1.55E-07	0.7	0	-	0.86	0	-	0.25568	32.87099	84	0.20822	0.09158	0.12116	129.79827
Rh_FR_SNP116	2	71.1	2.06E-07	0.68	0	-	0.87	0	-	0.26374	31.89404	87	0.20645	0.11863	0.08173	133.98335
Rh_FR_SNP108	2	71.1	2.14E-07	0	0.57	1.4	0	0.64	1.7	0.50909	18.49907	86	0.23336	0.09997	0.13445	139.80505

Rh_FR_SNP115	2	71.1	5.04E-07	0.65	0	-	0.83	0	-	0.24432	29.71024	84	0.19367	0.10029	0.09927	125.03145
Rh_FR_SNP109	2	71.1	5.05E-07	-	0	0.68	-	0	0.85	0.66418	29.58948	86	0.19209	0.07862	0.18120	143.47759
Rh_FR_SNP110	2	71.1	7.70E-07	-	0	0.65	-	0	0.85	0.65217	28.44137	87	0.18496	0.11940	0.08105	133.85932
Rh_FR_SNP111	2	71.1	1.24E-06	0.84	0	-	1.06	0	-	0.29310	27.41155	83	0.19675	0.12803	0.11988	142.15625
Rh_FR_SNP112	2	71.1	1.54E-06	1.54	0.7	0	1.6	0.54	0	0.31288	15.96163	79	0.21570	0.11801	0.13047	133.98968
Rh_FR_SNP119	2	71.2	1.86E-08	0	0.51	1.48	0	0.43	1.72	0.58209	22.48695	80	0.26159	0.05862	0.19449	129.02518
Rh_FR_SNP120	2	71.3	4.23E-09	0.79	0	-	-	0.98	0	0.25269	42.54233	89	0.24312	0.06990	0.16723	138.84500
Rh_FR_SNP121	2	71.3	7.75E-08	0.79	0	-	0.96	0	-	0.22289	35.10367	80	0.22794	0.07438	0.17661	129.99559
Rh_FR_SNP123	2	71.6	7.65E-09	-	0	0.8	-	0	0.99	0.66176	41.03591	87	0.24659	0.06605	0.17661	137.03683
Rh_FR_SNP122	2	71.6	2.32E-08	-	0	0.81	-	0	1	0.66667	38.24797	83	0.24804	0.08064	0.16277	133.43321
Rh_FR_SNP124	2	71.8	1.88E-10	0.95	0	-	1.25	0	-	0.3125	54.29430	76	0.24872	2.34E-06	0.23442	105.04948
Rh_FR_SNP125	2	71.8	1.19E-09	0.92	0	-	1.15	0	-	0.28659	47.85226	78	0.30637	0.09811	0.11685	121.41144
Rh_FR_SNP126	2	72	6.51E-07	-	0	0.76	-	0	0.93	0.63265	28.72202	90	0.18396	0.09500	0.15678	150.14887
Rh_FR_SNP134	2	72.3	8.72E-12	-	0	0.9	-	0	1.14	0.66667	62.74403	85	0.30908	0.00975	0.24317	125.25879
Rh_FR_SNP132	2	72.3	6.34E-10	0.92	0	-	1.22	0	-	0.28090	48.73638	85	0.26628	0.02389	0.23953	131.89991
Rh_FR_SNP131	2	72.3	4.40E-09	0.91	0	-	1.21	0	-	0.25625	43.90973	77	0.25232	0.03870	0.19708	113.89254
Rh_FR_SNP128	2	72.3	8.24E-09	-	0	0.81	-	0	0.97	0.66165	41.02967	85	0.21964	3.03E-06	0.30260	137.97491
Rh_FR_SNP133	2	72.3	1.05E-08	-	0	0.76	-	0	1	0.66418	40.22189	86	0.23133	0.09148	0.10657	127.15230
Rh_FR_SNP129	2	72.3	3.43E-07	0.71	0	-	0.84	0	-	0.25543	30.48626	88	0.18019	0.02608	0.28904	152.55182
Rh_FR_SNP127	2	72.3	5.28E-07	-	0	0.69	-	0	0.85	0.64626	29.16017	92	0.17625	0.07138	0.19730	154.76846
Rh_FR_SNP130	2	72.3	9.11E-07	0.7	0	-	0.83	0	-	0.26630	27.95934	88	0.17258	0.04249	0.26089	152.49368
Rh_FR_SNP135	2	72.4	4.64E-09	-	0	0.91	-	0	1.24	0.64865	45.06588	69	0.29184	0.08320	0.06444	85.16013
Rh_FR_SNP136	2	72.4	3.23E-07	-	0	0.91	-	0	1.26	0.28977	30.89259	84	0.19675	0.03495	0.27187	145.32536
Rh_FR_SNP139	2	72.5	3.39E-11	1.04	0	-	1.33	0	-	0.28125	60.23695	76	0.36469	0.11111	0.06923	110.70580
Rh_FR_SNP138	2	72.5	5.60E-11	1.53	0.45	0	1.81	0.39	0	0.31847	32.87388	76	0.34506	0.08992	0.09538	109.41951
Rh_FR_SNP137	2	72.5	1.27E-10	-	0	0.98	-	0	1.28	0.63704	54.17845	83	0.31003	0.10725	0.05505	109.90807
Rh_FR_SNP142	2	72.6	4.66E-13	-	0	0.95	-	0	1.2	0.66912	72.31188	88	0.32699	0.02909	0.18926	123.27256
Rh_FR_SNP146	2	72.6	3.78E-12	-	0	0.88	-	0	1.13	0.65957	64.47222	90	0.29811	0.02093	0.21782	131.53215
Rh_FR_SNP144	2	72.6	3.07E-11	0.9	0	-	1.13	0	-	0.22561	60.07126	78	0.28757	0.00469	0.24118	112.59343
Rh_FR_SNP141	2	72.6	7.69E-11	-	0	0.86	-	0	1.12	0.66187	54.74402	89	0.28028	0.04678	0.18590	132.79756
Rh_FR_SNP140	2	72.6	5.57E-10	-	0	0.82	-	0	1.09	0.65926	48.98786	86	0.27672	0.09219	0.09376	123.69112
Rh_FR_SNP143	2	72.6	2.82E-09	-	0	0.76	-	0	1.02	0.68644	45.13802	78	0.26475	0.06470	0.09876	97.69155
Rh_FR_SNP145	2	72.6	4.81E-07	0.98	0	-	1.26	0	-	0.13710	32.24373	58	0.28689	0.05605	0.19891	92.08124
Rh_FR_SNP147	2	72.7	6.20E-19	1.25	0	-	1.23	0	-	0.41667	163.87127	62	0.43942	0.18541	0.00486	99.73949
Rh_FR_SNP150	2	72.7	2.05E-11	-	0	0.9	-	0	1.17	0.67424	59.61982	86	0.31145	0.03707	0.19025	125.36550
Rh_FR_SNP149	2	72.7	1.16E-10	0.85	0	-	1.13	0	-	0.25275	53.77134	87	0.28141	0.07285	0.13260	126.17484
Rh_FR_SNP148	2	72.7	1.95E-09	0.85	0	-	1.09	0	-	0.22785	46.79502	75	0.30161	0.13033	0.01236	93.95464
Rh_FR_SNP151	2	72.8	4.11E-49	-0.33	-0.21	0	-0.43	-0.41	0	0.45614	1392.559	58	0.88688	0.23961	2.40E-06	106.82612
Rh_FR_SNP160	2	72.8	6.64E-13	0.95	0	-	1.2	0	-	0.25543	71.03466	88	0.31044	2.52E-06	0.25163	127.86169

Rh_FR_SNP152	2	72.8	1.44E-12	-	0	0.93	-	0	1.19	0.66667	68.02521	89	0.31463	0.02841	0.19879	126.95835
Rh_FR_SNP153	2	72.8	2.51E-12	-	0	0.93	-	0	1.19	0.66667	66.10609	89	0.31112	0.03078	0.19745	127.89891
Rh_FR_SNP159	2	72.8	4.29E-10	0.8	0	-	1.07	0	-	0.25	50.10519	84	0.25320	0.03630	0.15980	111.41582
Rh_FR_SNP155	2	72.8	2.82E-09	-	0	0.83	-	0	1.13	0.64706	44.18746	85	0.25396	0.03553	0.23156	134.90576
Rh_FR_SNP158	2	72.8	4.48E-09	-	0	0.89	-	0	1.11	0.69027	44.00323	76	0.25736	0.02182	0.26313	123.58101
Rh_FR_SNP154	2	72.8	1.87E-08	0.9	0	-	1.2	0	-	0.27381	39.18917	80	0.25787	0.08245	0.16093	130.31732
Rh_FR_SNP156	2	72.8	7.73E-08	-	0	0.67	-	0	1	0.66667	35.59680	75	0.24933	0.11523	0.04733	99.11055
Rh_FR_SNP157	2	72.8	7.49E-07	-	0	0.7	-	0	0.91	0.69725	29.39573	73	0.17822	0.02085	0.21614	104.95468
Rh_FR_SNP161	2	72.9	2.66E-10	0	0.3	1.33	0	0.21	1.59	0.58741	28.99573	85	0.31398	0.11567	0.04601	112.96186
Rh_FR_SNP165	2	72.9	8.05E-10	-	0	0.84	-	0	1.04	0.65714	47.45656	89	0.24137	2.94E-06	0.29387	141.96850
Rh_FR_SNP164	2	72.9	1.75E-09	-	0	0.84	-	0	1.04	0.66418	45.49512	86	0.24810	2.98E-06	0.29821	139.37934
Rh_FR_SNP163	2	72.9	2.76E-09	0.81	0	-	1.02	0	-	0.25275	44.02123	87	0.21938	2.94E-06	0.29352	138.63979
Rh_FR_SNP162	2	72.9	7.01E-09	-	0	0.97	-	0	1.35	0.62143	41.61253	84	0.26256	0.09220	0.13973	135.66216
Rh_FR_SNP166	2	73	3.61E-10	-	0	0.97	-	0	1.29	0.63309	50.49035	85	0.28892	0.05809	0.17278	128.90261
Rh_FR_SNP167	2	73	1.73E-09	-	0	0.93	-	0	1.22	0.62759	45.28156	88	0.26057	0.06840	0.16304	136.07973
Rh_FR_SNP170	2	73	8.36E-09	0.83	0	-	1.01	0	-	0.26437	41.09490	84	0.22653	3.14E-06	0.31359	140.29947
Rh_FR_SNP175	2	73	9.28E-09	-	0	0.88	-	0	1.03	0.67480	41.24719	80	0.22335	3.09E-06	0.30855	132.30665
Rh_FR_SNP172	2	73	1.17E-08	0	0.58	1.48	0	0.5	1.71	0.56757	22.75819	87	0.25539	0.08210	0.14144	134.66521
Rh_FR_SNP169	2	73	2.76E-08	0.89	0	-	1.16	0	-	0.31818	38.85762	73	0.26026	0.13750	0.01321	95.61955
Rh_FR_SNP168	2	73	4.47E-08	-	0	0.82	-	0	0.99	0.67213	36.76405	79	0.23520	0.01733	0.29315	133.44375
Rh_FR_SNP173	2	73	6.32E-08	0	0.59	1.48	0	0.46	1.67	0.57333	20.17749	88	0.23690	0.09554	0.13515	142.00536
Rh_FR_SNP174	2	73	2.75E-07	-	0	0.73	-	0	0.85	0.68333	31.67255	79	0.19084	3.11E-06	0.31119	130.30300
Rh_FR_SNP171	2	73	7.05E-07	-	0	0.74	-	0	0.9	0.67769	29.13581	79	0.18148	3.20E-06	0.31970	132.40568
Rh_FR_SNP179	2	73.1	1.09E-08	-	0	0.83	-	0	1.1	0.68750	41.55467	74	0.28512	0.09677	0.09728	108.42398
Rh_FR_SNP177	2	73.1	1.11E-08	0.79	0	-	0.98	0	-	0.27778	40.05845	86	0.22457	0.00241	0.30614	142.77827
Rh_FR_SNP178	2	73.1	1.40E-07	1.12	0.15	0	1.36	0.12	0	0.31034	19.80777	73	0.25908	0.12744	0.04025	99.69293
Rh_FR_SNP176	2	73.1	3.23E-07	0.76	0	-	0.98	0	-	0.65421	32.31039	67	0.25240	0.07097	0.17295	106.60755
Rh_FR_SNP182	2	73.2	3.01E-09	-	0	0.82	-	0	0.99	0.66176	43.76106	87	0.23117	3.03E-06	0.30258	141.25338
Rh_FR_SNP181	2	73.2	5.53E-09	0.78	0	-	0.93	0	-	0.26316	41.57361	91	0.21542	3.03E-06	0.30308	147.97320
Rh_FR_SNP184	2	73.2	4.07E-08	-	0	0.76	-	0	0.94	0.67717	36.64724	83	0.19966	2.91E-06	0.29145	131.61135
Rh_FR_SNP180	2	73.2	1.52E-07	0.77	0	-	0.93	0	-	0.25294	33.14624	81	0.19487	0.01618	0.27533	131.59465
Rh_FR_SNP183	2	73.2	2.00E-07	-	0	0.76	-	0	0.89	0.68033	32.47467	80	0.19926	3.15E-06	0.31549	134.06365
Rh_FR_SNP185	2	73.3	1.21E-09	-	0	0.89	-	0	1.1	0.70476	49.11829	71	0.27959	0.07620	0.10737	97.35702
Rh_FR_SNP190	2	73.3	2.59E-08	-	0	0.73	-	0	1	0.67164	37.56688	87	0.23003	0.09222	0.11174	129.19441
Rh_FR_SNP186	2	73.3	4.44E-08	-	0	0.75	-	0	1	0.68595	36.68212	80	0.24619	0.09569	0.10083	117.82347
Rh_FR_SNP189	2	73.3	9.76E-08	0.76	0	-	0.98	0	-	0.27222	33.97661	86	0.21346	0.05575	0.21485	142.28384
Rh_FR_SNP188	2	73.3	1.69E-07	1.22	0.28	0	1.44	0.16	0	0.33129	19.02382	82	0.24055	0.10278	0.13304	132.68007
Rh_FR_SNP187	2	73.3	3.04E-07	-	0	0.78	-	0	0.93	0.69298	31.63191	76	0.17833	3.07E-06	0.30695	125.26785
Rh_FR_SNP191	2	73.3	4.68E-07	-	0	0.68	-	0	0.87	0.66917	29.79050	86	0.16312	0.00593	0.29254	140.69665

Rh_FR_SNP192	2	73.4	1.96E-07	0.75	0	-	0.94	0	-	0.22989	32.30651	83	0.19193	0.01605	0.28647	137.67042
Rh_FR_SNP193	2	73.4	1.33E-06	0.92	0.17	0	1.11	0.08	0	0.26144	16.38175	74	0.21677	0.08980	0.11890	111.76760
Rh_FR_SNP197	2	73.5	2.96E-07	-	0	0.73	-	0	0.9	0.67164	30.93136	87	0.18768	0.02567	0.28749	149.08067
Rh_FR_SNP196	2	73.5	2.98E-07	-	0	0.71	-	0	0.89	0.66667	30.79841	89	0.18340	0.02651	0.28093	151.20740
Rh_FR_SNP195	2	73.5	4.64E-07	-	0	0.7	-	0	0.9	0.66187	29.64457	89	0.18886	0.05342	0.22728	149.12329
Rh_FR_SNP194	2	73.5	5.99E-07	-	0	0.69	-	0	0.9	0.66187	28.98649	89	0.18641	0.06573	0.20586	148.43425
Rh_FR_SNP199	2	73.7	1.40E-08	1.24	0.24	0	1.56	0.26	0	0.33962	22.86509	81	0.26370	0.06495	0.16664	123.24693
Rh_FR_SNP198	2	73.7	1.91E-07	1.22	0.31	0	1.42	0.26	0	0.34091	18.61981	87	0.22062	0.07610	0.16086	136.67191
Rh_FR_SNP200	3	6.8	9.89E-08	0	0.48	1.6	0	0.68	2.05	0.48193	19.81364	82	0.25530	0.16076	0.06303	135.41198
Rh_FR_SNP201	3	7.3	4.27E-11	0	-1.21	-	0	-1.25	-	0.43671	59.68497	75	0.35107	0.16990	0.02489	115.58685
Rh_FR_SNP203	3	8.9	1.45E-10	-	-1.32	0	-	-1.37	0	0.53636	62.33968	55	0.42908	0.18392	0.02410	85.34578
Rh_FR_SNP202	3	8.9	8.58E-08	1.51	0.35	0	1.85	0.4	0	0.475	20.13486	80	0.27374	0.14675	0.06062	127.07123
Rh_FR_SNP204	3	9.2	6.62E-07	0	0.55	1.63	0	0.71	2.02	0.49342	17.34977	75	0.25378	0.14224	0.08036	122.71587
Rh_FR_SNP205	3	9.9	1.83E-07	0	0.47	1.59	0	0.62	2.01	0.48765	19.00501	80	0.25086	0.14510	0.06920	129.28866
Rh_FR_SNP206	3	10.4	5.78E-07	0	0.23	1.45	0	0.19	1.69	0.48538	17.16325	84	0.23155	0.17702	0.05925	145.63252
Rh_FR_SNP207	3	12.3	5.31E-07	0	-1.1	-1.33	0	-1.43	-1.75	0.51299	17.40264	81	0.23456	0.14466	0.07136	132.19418
Rh_FR_SNP208	3	12.3	1.08E-06	0	-1.09	-1.36	0	-1.42	-1.83	0.50658	16.47311	79	0.23393	0.16378	0.07151	135.38432
Rh_FR_SNP209	3	12.4	9.41E-07	0	-1.12	-1.37	0	-1.17	-1.56	0.55556	16.83645	75	0.24853	0.16769	0.06209	123.60556
Rh_FR_SNP210	4	47.5	2.11E-07	-	1.06	0	-	0.54	0	0.53061	32.73330	75	0.23335	0.23793	0.03626	144.06074
Rh_FR_SNP211	5	3.7	1.66E-06	-	0	0.69	-	0	0.71	0.58503	26.66544	83	0.18713	0.18156	0.04125	137.07428
Rh_FR_SNP212	5	13.2	2.32E-07	-0.84	0	-	-0.87	0	-	0.09375	32.38634	76	0.21729	0.16909	0.03552	126.12204
Rh_FR_SNP213	5	55.9	7.78E-84	-	0.44	0	-	0.29	0	0.67368	35809.117	60	0.75329	0.21454	2.15E-06	103.87214
Rh_FR_SNP214	5	73.9	7.89E-53	-	0.12	0	-	0.04	0	0.55172	3131.007	60	0.75267	0.21610	2.16E-06	100.16309
Rh_FR_SNP215	7	27.2	1.03E-12	-0.12	-0.21	0	0.16	-0.14	0	0.44094	43.82248	65	0.44181	0.24352	7.76E-05	111.36610
Rh_FR_SNP216	7	28.8	8.79E-13	0	-0.74	-1.35	0	-0.09	-0.57	0.45562	39.51273	84	0.37987	0.25315	0.01584	150.63016
Rh_FR_SNP217	7	40.9	1.62E-42	0	0.1	-	0	-0.13	-	0.28571	1987.216	52	0.90520	0.23420	2.34E-06	100.67248
Rh_FR_SNP218	7	65.4	2.94E-11	0	-1.28	-1.21	0	-0.81	-0.91	0.54967	32.94340	84	0.34112	0.21710	0.02028	147.11140

[1)] The effect of the genotypic class with the lowest frequency is set to zero. The effects of the other genotypic classes are given as deviations between their estimated values and the lowest frequency class.
